# Structured generative modelling of earthquake response spectra with hierarchical latent variables in hyperbolic geometry

**DOI:** 10.1038/s41598-025-29902-6

**Published:** 2026-01-07

**Authors:** Alfred Wright, Jawad Fayaz

**Affiliations:** Department of Computer Science, University of Exeter, EX4 4RN Exeter, United Kingdom

**Keywords:** Generative AI, Hierarchical variational autoencoder, Response spectra, Early warning systems, Latent variable modelling, Stochastic ground motion simulation, Poincaré ball, Engineering, Mathematics and computing, Solid Earth sciences

## Abstract

This study presents a geometry-aware generative modelling framework for earthquake response spectra, leveraging a hierarchical variational autoencoder (HVAE) with latent variables embedded in a Poincaré ball manifold. Predicting complete ground motion response spectra is crucial for seismic hazard analysis and structural performance assessment; however, conventional machine learning models often fail to capture multi-scale physical dependencies and hierarchical uncertainity inherent in earthquake records that arise from event-to-event variablity and spatial variability. The proposed architecture is trained using source and site parameters to regularise the latent space which enables the generation of physically consistent spectral amplitudes while explicitly modelling inter- and intra-event variabilities . By exploiting hyperbolic latent geometry, the HVAE encodes hierarchical relationships into the latent space with an improved representational efficiency. Trained on a curated strong-motion dataset, the model achieves high reconstruction fidelity, with a mean coefficient of determination of 0.961 across all spectral periods. Integration into stochastic ground motion simulation and early warning pipelines demonstrates its practical utility. This work bridges geometric deep learning and seismological modelling, offering a principled, domain-aligned approach to real-time seismic risk mitigation.

## Introduction

Earthquakes are among the most devastating natural hazards, accounting for a significant portion of global disaster fatalities. Between 1998 and 2017, over 750,000 people died and more than 125 million were displaced or injured due to earthquakes^1^. Their impact is profound, disrupting not just communities but also economic, transportation, and energy systems^2–6^. Accurate modelling and forecasting of earthquake-induced ground motion remains a high priority across disciplines, underpinning resilient urban design, infrastructure planning, and real-time emergency response^7,8^. Central to this effort is the deployment of earthquake early warning systems (EEWS), which estimate shaking intensity before strong ground motion arrives^9,10^. The success of such systems depends on fast and reliable models capable of predicting key ground motion characteristics within seconds of earthquake initiation.

A core metric in these systems is the response spectrum ($S_a(T)$), which represents peak acceleration response over a range of vibration periods (*T*). It is fundamental to seismic hazard assessment, building code formulation, and performance-based engineering^11^. Given its strong physical basis and interpretability, $S_a(T)$ is widely adopted as a target in both empirical and data-driven ground motion prediction efforts. Traditional approaches such as ground motion models (GMMs) express $S_a(T)$ as a deterministic function of source, path, and site parameters^12,13^, but are constrained by strong assumptions and limited flexibility. Historically, studies often treated the two horizontal/orthogonal components of ground motions separately to compute $S_a(T)$. However, this led to a non-unique relationship between earthquake parameters and spectral ordinates because the horizontal amplitudes depend on the arbitrary recording direction. To mitigate this, researchers explored component-combining measures such as the arithmetic mean, geometric mean, and square-root-sum-of-squares. These metrics improved stability but still lacked rotational invariance^14^. The current state of practice therefore adopts RotD50 $S_a(T)$–defined as the 50th-percentile spectral acceleration computed across all possible rotation angles of the horizontal components^11^. RotD50 provides an orientation-independent, physically interpretable descriptor of shaking and is now the standard horizontal spectral measure in GMMs and modern early-warning and hazard frameworks. For these reasons, this study also relies on RotD50 component as the $S_a(T)$.

Machine learning (ML) and deep learning (DL) methods have been extensively adopted across a range of seismological and EEWS tasks. These include earthquake detection and classification^15,16^, hypocentre localisation^17,18^, magnitude estimation^19^, and seismic phase picking^20^. In ground motion modelling, ML methods have enhanced predictions of peak ground motion metrics^21^, site response functions^22,23^, and spectral amplitudes^24,25^. Several studies have also integrated ML into probabilistic seismic hazard analysis (PSHA) workflows^26,27^, and EEWS frameworks^28–30^, including real-time Bayesian updating with latent embeddings^25^.

While ML has delivered measurable success in various seismological tasks, its application to ground motion simulation–particularly within PSHA and EEWS–remains largely restricted to deterministic or point-estimate models without uncertainty quantification or generative capabilities. Typically, these models map source-site parameters (e.g., magnitude, distance) to scalar ground motion metrics such as peak ground acceleration (PGA) or $S_a(T)$^24^, but they often overlook aleatoric uncertainty, spectral correlations, and structured hierarchical variability across stations and events. Such limitations are critical in applications like PSHA, where hazard curves depend on the full distribution of plausible ground motions, or EEWS, where robust posterior updates require uncertainty-aware forward models^25,30^. In practice, success depends not only on prediction accuracy but also on simulation, scenario generation, and model interpretability.

These challenges are mirrored across other geophysical hazards and climate modelling domains, where ML has emerged as a powerful tool for predictive modelling and risk assessment. Applications include landslide prediction and susceptibility analysis^31,32^, wildfire spread^33^, and flood forecasting^34^. Recent work in climate science has demonstrated the potential of data-driven models for event-based detection, scenario exploration, and uncertainty quantification^35,36^. This has emphasised that many of these domains now require models that go beyond regression: they must generate coherent scenarios under uncertainty, interpolate between observations, and support interpretable exploration of physical relationships.

This convergence of generative needs has motivated the rise of generative artificial intelligence (GenAI), with foundation models such as variational autoencoders (VAEs)^37^, generative adversarial networks (GANs)^38^, and diffusion models^39^ enabling high-quality sample generation from learned distributions. These models learn high-dimensional distributions and enable probabilistic sampling of plausible data. Among these, VAEs remain particularly well-suited for scientific domains due to their probabilistic formulation, explicit latent structure, and capacity for uncertainty-aware simulation. Such models mirror the demands of ground motion analysis, where one must generate site-specific spectra conditioned on uncertain inputs.

Yet, the uptake of GenAI in seismology has been limited. While recent studies have adopted VAEs for learning latent representations of spectra^25^ and waveform-to-intensity mappings^40^, they treat ground motions as flat identically and independently distributed (IID) samples, neglecting the fact that recordings are inherently grouped by earthquake events or stations. Furthermore, traditional Euclidean latent spaces used in VAEs poorly reflect the hierarchical or tree-like nature of these relationships. These shortcomings are particularly evident in seismology, where data is naturally grouped (e.g., multiple recordings from the same earthquake) and $S_a(T)$ spectra exhibit structured cross-period dependencies. These limitations reduce their interpretability and generalisation when applied to physical earthquake-related systems.

Without architectural adjustments, existing generative models struggle to produce diverse yet physically consistent ground motion information. This disconnect between the structured nature of seismic data and the design of generative models limits their utility in tasks such as stochastic simulation, EEW, operational forecasting, and uncertainty-aware hazard mapping. Latent space modelling is a key component of any generative framework, and the design of this space can greatly affect model performance and interpretability. Mixed-effects modelling^12,13,41^ has long been used in statistics to model grouped data, separating fixed and random effects. Recent efforts have sought to integrate these ideas into deep generative models by encoding group structure through separate latent variables or group-conditioned priors^42^. However, these approaches are often computationally expensive and do not scale well with fine-grained or nested hierarchies. An alternative strategy comes from geometric DL, where latent spaces are embedded in non-Euclidean manifolds. This approach introduces curvature into the latent space through hyperbolic geometry, providing a mathematically principled mechanism for representing hierarchical structures. Unlike traditional mixed-effects formulations, which impose hierarchy through additive statistical terms, the hyperbolic latent manifold embeds hierarchical relationships directly into the geometry itself. This enables the model to more faithfully capture the multi-level structure arising from multiple recordings across multiple earthquakes, yielding a representation that is both physically informed and structurally coherent.

Hyperbolic geometry,particularly the Poincaré ball model, offers a mathematically principled framework for embedding data with hierarchical or tree-like structure^43^. Unlike Euclidean space, where distance scales linearly, hyperbolic space exhibits exponential volume growth, making it ideal for representing datasets where the number of elements increases multiplicatively across levels, such as taxonomies or spatio-temporal hierarchies. In the Poincaré ball model, distances expand rapidly toward the boundary, enabling efficient encoding of fine-grained distinctions at deeper levels of the hierarchy. This property has been exploited in domains such as knowledge graph completion^44^, natural language inference^45^, and biological taxonomy classification^46^. Integrating hyperbolic geometry into the latent space of generative models can facilitate more compact representations, reduce distortion of hierarchical relationships, and yield latent embeddings that better reflect the physical and statistical structure of the underlying phenomena^47,48^.

Hierarchical generative modelling for structured and multi-scale scientific data poses distinct challenges that are not adequately addressed by conventional architectures. Recent efforts to mitigate this have explored mixed-effects VAEs^42,49^, group-conditioned priors^50^, or multi-level encoders^51^, but these approaches are computationally intensive and often assume Euclidean latent priors. Moreover, the role of latent geometry remains largely unexamined, despite its potential to encode domain-specific inductive biases such as spatial locality, scale separation, or hierarchical composition^47,48,52^. For seismic ground motion data, intra-event variability arises from local site effects and wave propagation paths, while inter-event differences reflect source properties and regional tectonics. A generative model that explicitly captures this dual structure–via hierarchical conditioning and geometrically consistent latent spaces–can offer both physical interpretability and practical utility for downstream tasks.

$S_a(T)$ provides an ideal testbed for evaluating such structured generative models. $S_a(T)$ spectra are high-dimensional, continuous-valued, and strongly correlated across periods *T*. Spectral response curves reflect both event-level (inter-event) properties (e.g., rupture mechanism, magnitude) and site-specific (intra-event) modifications (e.g., soil shear-wave velocity, basin effects), motivating a two-level latent space where coarse features are captured at the event level and fine details at the station level. Embedding these latent variables in a hyperbolic space further allows the model to reflect nested dependencies more efficiently, while maintaining smooth interpolation and regularisation across related samples.

This study develops a three-stage generative modelling framework for earthquake ${S_a}(T)$ spectra, grounded in hierarchical and geometric latent representations. At the core of the framework is a hierarchical VAE (HVAE) that learns multiscale latent embeddings of ${S_a}(T)$, explicitly structured by event identity to disentangle inter-event and intra-event variability. The latent structure is embedded within a Poincaré ball to exploit hyperbolic geometry for capturing nested variability. The HVAE’s latent space is tuned using moment magnitude (*M*), hypocentre depth ($Z_{\textrm{hyp}}$), rupture source-to-site distance ($R_{\textrm{rup}}$), and soil’s average shear-wave velocity at the topmost 30m ($V_{s30}$), enabling physically consistent and site-specific generation of $S_{a}(T)$ spectra. This component forms the backbone for all subsequent modules. The HVAE is then trained and tested for two downstream applications of EEW and stochastic ground motion simulation.

For EEW, a feed-forward neural network (FFNN) is trained to regress from partial intensity measures (IMs) computed from early waves after detection of seismic P-waves at a given station to the latent space of the HVAE. This allows decoding of likely spectral shapes and amplitudes before the full ground motion waveform arrives at a given station. The FFNN acts as a rapid encoder that maps sparse IMs into a distributional latent representation, which is then decoded by the HVAE-decoder to estimate full $S_a(T)$ spectra. For stochastic ground motion simulation, a conditional VAE (CVAE) is trained independently which learns a direct generative mapping from earthquake parameters (*M*, $Z_{\textrm{hyp}}$, $R_{\textrm{rup}}$, $V_{s30}$) to the HVAE’s latent space, enabling posterior sampling under input uncertainty. The samples are then used to generate candidate $S_a(T)$ which can then be used as targets for spectral matching by previously developed stochastic ground motion simulation tools. By leveraging the probabilistic nature of both HVAE and CVAE latent representations, the framework provides a principled approach for uncertainty-aware simulation consistent with GenAI paradigms. Hence, this work contributes a generative modelling framework that bridges ML, geometric DL, and earthquake engineering, enabling interpretable, hierarchical, and physically grounded synthesis of $S_a(T)$ spectra.

## Results

This study presents a structured generative modelling framework for synthesising earthquake $S_a(T)$ spectra, grounded in hierarchical latent representations and process-informed learning. The central aim is to model the inter- and intra-event variability of $S_a(T)$ spectra while maintaining physical interpretability, geometric coherence, and sampling flexibility. The results are structured in four parts: (1) the conceptual and architectural foundation of the generative HVAE model, (2) evaluation and exploration of the generative model capabilities, (3) its deployment in early warning scenarios using partial observations, and (4) its use in forward ground motion simulation under conditioning inputs.

### Framework conceptualisation

This study presents a hierarchical generative framework for modelling earthquake $S_a(T)$ spectra by learning structured latent representations that account for both physical dependencies and data hierarchies. The proposed method is anchored in a VAE^37^ design with a nested prior architecture and non-Euclidean geometry, tailored for structured seismic data and constrained by domain-specific inductive priors. The central premise is that seismic recordings are not IID but are instead structured hierarchically: multiple recordings (station-level observations) are nested within earthquake events. To reflect this, the proposed model constructs a hierarchical prior over the regularised latent space of a VAE, enabling the joint encoding of inter-event and intra-event variability through a two-stage generative process.

**Fig. 1:**
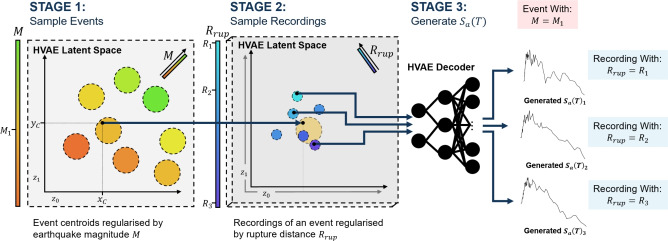
Schematic of the two-stage sampling approach followed by the generation stage. Stage 1 involves sampling an event centroid from the latent space $(x_C, y_C)$, noting estimated event-level attributes such as magnitude $M=M_1$. Stage 2 involves sampling a recording in the latent space surrounding $(x_C, y_C)$, noting estimated recording-specific site attributes such as rupture distance $R_{\textrm{rup}}=R_1$. Stage 3 involves passing the sampled latent variables to the HVAE-decoder to generate a $S_a(T)$ spectrum. Multiple samples can be taken surrounding $(x_C, y_C)$ to generate spectra from multiple stations.

At the core of the framework is the HVAE with a hierarchical latent variable model that captures multi-scale variability in ground motion recordings. Specifically, a two-level latent structure is imposed: (i) inter-event latent variables conditioned on earthquake-level attributes (e.g., *M*), and (ii) intra-event latent variables conditioned on recording-specific site attributes (e.g., $R_{\textrm{rup}}$). The nested latent space architecture implements partial pooling by regularising the station-level embeddings towards the corresponding event-level centroids, encoding structured correlations between recordings of the same earthquake while preserving fine-grained local variability. This hierarchical structure is embedded within a Poincaré ball manifold^47^, allowing the model to exploit the exponential expansion of hyperbolic geometry to represent nested relationships efficiently. The latent structure thus enforces both physical interpretability and hierarchical coherence across samples. The construction of $S_a(T)$ proceeds via a trained decoder that maps these hierarchically sampled latent representations to $S_a(T)$ across periods (*T*), as illustrated in Fig. 1.

In addition to its architectural novelty, the model introduces a suite of hierarchical and feature-informed regularisation terms that go beyond traditional reconstruction and Kullback-Leibler (KL) divergence^53^ terms. These include: i) a KL-divergence term between each sample embedding and its associated event-level prior; ii) a KL-divergence term between each event-level prior and a shared hyper-prior; iii) a feature-based regularisation of sample embeddings using recording-specific station-level features; and iv) a regularisation of event-level priors using event-level features. These loss components impose a hierarchical structure and promote physically meaningful organisation in the latent space. The overall objective thus balances data fidelity, latent compression, and physical interpretability.

Together, the hierarchical prior, hyperbolic embedding, and physically structured losses allow the model to generate site-specific spectral predictions that are both geometrically and physically plausible. This latent structure also enables transfer across tasks in this study: an FFNN maps early-time P-wave-derived IMs to the latent space for anticipatory inference, while a CVAE maps source-site parameters directly to the hierarchical latent space for forward stochastic simulation. Each component is fully differentiable and trained independently but shares the core geometry and latent structure defined by the HVAE.

### Spectral fidelity and generative capabilities of the HVAE

#### Reconstruction accuracy and multi-period coherence

The fidelity of the HVAE model in reconstructing $S_a(T)$ spectra is quantitatively evaluated using two complementary metrics: the coefficient of determination ($R^2$)^54^ across *T* and the aggregated index of agreement (IoA)^55^ across samples. Together, these metrics capture both local reconstruction performance at individual *T* and global fidelity across the full spectral shape.

**Fig. 2:**
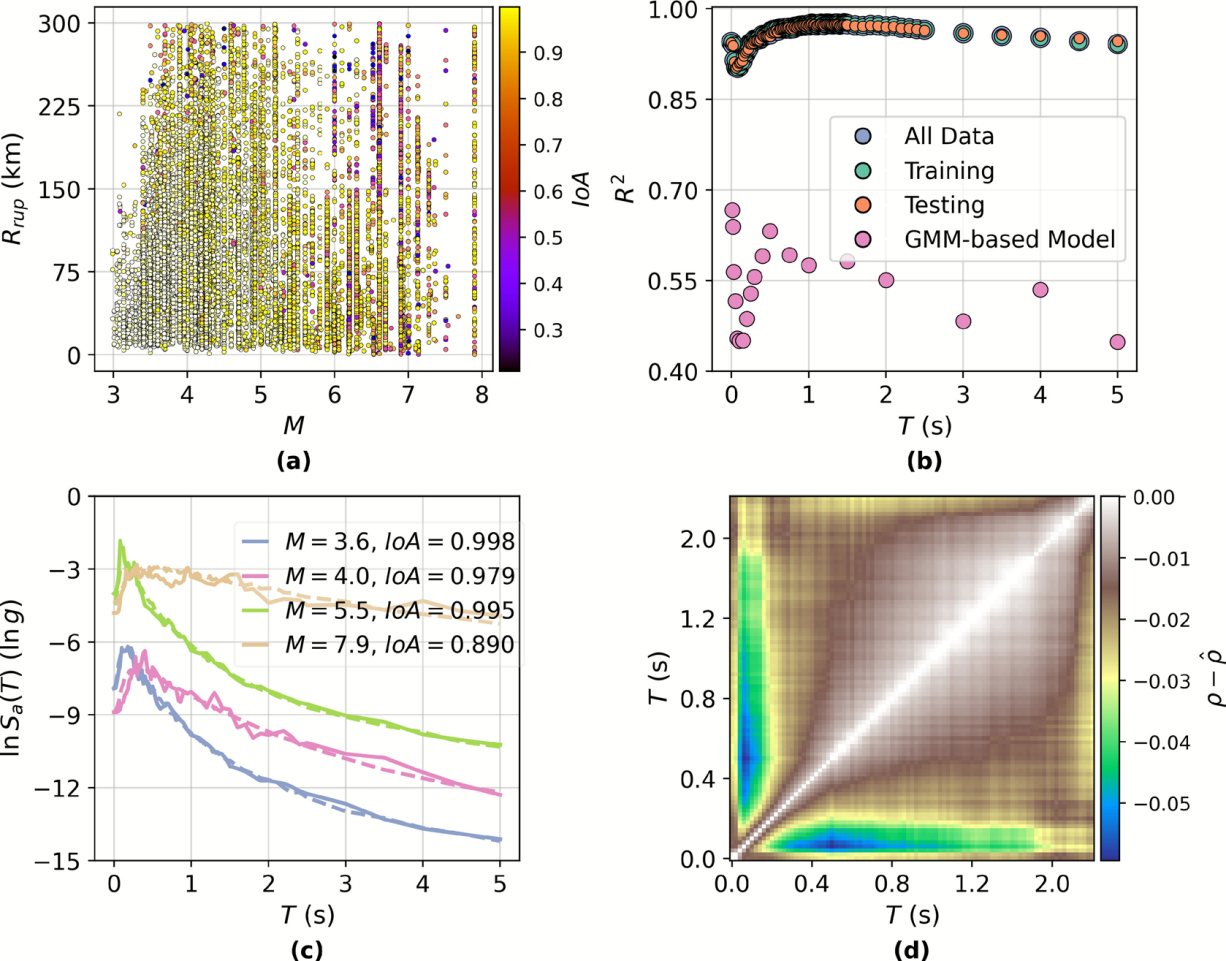
Reconstruction results. (**a**) A scatter plot of magnitude *M* against rupture distance $R_{\textrm{rup}}$ colour-coded by index of agreement (IoA). (**b**) A scatter plot of the coefficient of determination ($R^2$) against the response spectrum period. (**c**) Four example reconstructed response spectra, best-performing and median-performing by IoA for both lower *M* earthquakes and higher *M* earthquakes (solid line is original and dashed line is reconstructed). (**d**) A heat map of the difference in cross-correlation coefficients for the true response spectra $\rho$ and reconstructed response spectra ${\hat{\rho }}$ at each pair of spectral periods.

Figure 2b reports $R^2$ values computed across all *T* values, revealing that the HVAE achieves consistent performance, with values exceeding 0.9 at every *T* value and a mean $R^2$ of 0.961. The minimal difference between training and testing performance (mean $R^2$ of 0.961 and 0.960, respectively) suggests low over-fitting and strong generalisability. Performance remains robust across the *T* range, except for very low *T* (below 0.2 s), where reduced accuracy is observed due to amplified recording noise and limited structural energy content^56^. Notably, the model achieves high reconstruction accuracy ($R^2 >0.95$) for $T > 0.5$ s, which approximately corresponds to the natural periods of mid- to high-rise structures (e.g., $T \approx 0.1 \times$ number of storeys), a range that is of particular interest in engineering applications^57^. In general, although a total mean $R^2$ of 0.961 is indeed very strong, previous simple VAE approaches have achieved better^28^. The added complexity and physical considerations of the HVAE naturally creates a mild trade-off between performance and interpretability. However, this trade-off is well-justified given the physics-informed and hierarchical nature of the model, enabling more meaningful use of the data.

Figure 2a further explores the IoA distribution across events, allowing spatial and physical analysis of reconstruction errors. Events with lower *M* ($M\in [3.0, 4.0]$) and moderate $R_{\textrm{rup}}$ yield IoA scores above 0.9, indicating near-perfect spectral generation. In contrast, higher *M* events ($M\in [6.0, 8.0]$) exhibit increased intra-event spectral variability, leading to reduced agreement (mean IoA $\approx 0.80$). This degradation likely reflects a combination of physical complexity (e.g., increased rupture heterogeneity, basin effects) and data imbalance–high-*M* events are under-represented in the training set despite undersampling procedures. This difference in reconstruction performance is demonstrated with examples in Fig. 2c. The examples include two lower *M* samples and two higher *M* samples (split by $M=5$) with the best-performing and median-performing cases as per IoA. Both best-performing reconstructions are highly accurate in matching the amplitude and shape of the spectrum whilst the median-performance reconstructions show mild deviations. Importantly, the current data curation strategy reflects the real-world frequency distribution of events and thus prioritises modelling fidelity where operational deployment is most critical (i.e., frequent, moderate *M* events).

To evaluate the preservation of structural dependencies across *T* values, the difference in correlation matrices between the true and reconstructed response spectra is examined (Fig. 2d). Specifically, the Pearson correlation coefficients ($\rho$)^54^ computed across all pairs of *T* values for the observed $S_a(T)$ are compared against those for the reconstructed $S_a(T)$, (${\hat{\rho }}$), and the pairwise differences are visualised as a heatmap. The HVAE effectively maintains cross-*T* correlation structure, particularly for mid-to-long *T* ($T\in [0.5\ \text {s}, 2.0\ \text {s}]$), which are critical for engineering applications involving structural dynamics and seismic risk estimation^58^. The maximum observed discrepancy occurs between $T=0.06 \,\text {s}$ and $0.5\,\text {s}$, with a correlation difference of $-0.0594$, indicating limited distortion even at challenging low *T* ranges. These shorter *T* are known to exhibit greater sensitivity to recording noise and site-specific amplification effects^56^, which can degrade reconstruction fidelity. Importantly, the correlations at *T* relevant for performance-based earthquake engineering, such as $T \sim 1.0\,\text {s}$, corresponding to the fundamental period of mid-rise buildings^57^, are well preserved. This fidelity in reproducing the correlation structure ensures that generated $S_a(T)$ remain realistic not only in marginal distributions but also in joint behaviour, a requirement for reliable use in system-level design and PSHA.

While no direct one-to-one baselines exist for hierarchical DL models with statistical surrogacy and physics-informed constraints, the observed reconstruction fidelity is markedly higher than what is typically reported for conventional empirical GMMs^11–13^ and previous statistical surrogacy studies such as Fayaz and Galasso^28^. Furthermore, a brief analysis of a traditional GMM-based prediction scheme is undertaken using the Campbell-Bozorgnia (2014) GMM formulation^12^ with the available source and site inputs of the next-generation attenuation (NGA) west 2 database. As shown in Fig. 2b, the resulting $S_a(T)$ predictions exhibit substantial dispersion and systematic underestimation at both short and intermediate periods. This behaviour is consistent with the documented limitations of empirical GMMs leading to lower predictive power and not being able to capture cross-period spectral coherence^11,12^. It is observed that the GMM’s performance remains markedly lower across all periods (typically $R^2 \approx 0.45$–0.60), even with inputs of source parameters like *M* which is not an input to the HVAE. In contrast, the HVAE maintains $R^2 > 0.90$ across nearly the full period range, for both training and testing subsets, reflecting its ability to represent joint spectral structure and hierarchical variability. This comparison further highlights that the HVAE serves not merely as a predictive model but as a coherent generative surrogate, capable of reconstructing spectrum-wide features with substantially greater fidelity than conventional GMM approaches. Nevertheless, it is important to mention that this is the first study, to the authors’ knowledge, to couple a hierarchical latent-space architecture with physics-informed constraints for $S_a(T)$ modelling, making strict numerical comparisons with earlier empirical or shallow-learning approaches inappropriate.

It is evident that the model performance has some bias against higher *M* events, typically achieving lower IoA and slightly broader residuals when compared to lower *M* events. As discussed, a primary cause of this discrepancy is likely the physical complexity associated to higher *M* events such as more heterogeneous rupture processes, stronger directivity and path effects, and potential non-linear site responses, all of which can lead to greater variability in $S_a(T)$. By incorporating additional physical information, such as fault type, into latent space modelling, perhaps through additional layers in the hierarchy, and with additional data, it would be possible to reduce the bias further. Future research could explore this.

#### Generalisation across *M* and $R_{\textrm{rup}}$

**Fig. 3:**
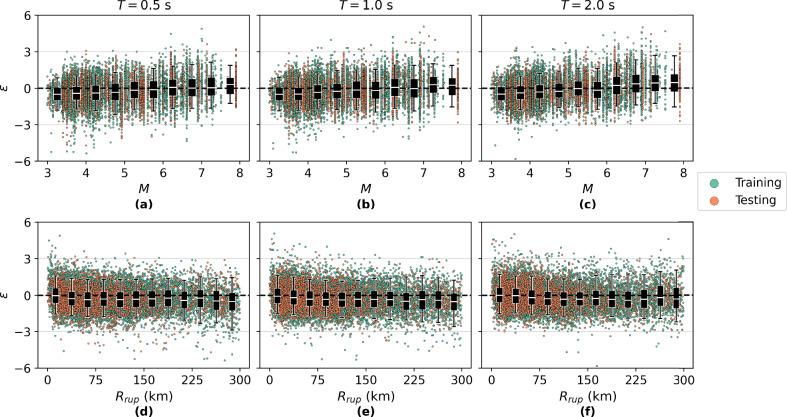
Residual analysis. Scatter plots of the residual, $\epsilon = \frac{\ln {S_a(T)} - \ln {\hat{S}_a(T)}}{\text {std}\left( \ln {S_a(T)} - \ln {\hat{S}_a(T)}\right) }$, from each sample by magnitude *M* and rupture distance $R_{\textrm{rup}}$ for periods $T\in [0.5\text { s}, 1.0\text { s}, 2.0\text { s}]$.

To further evaluate the generalisation performance of the HVAE across the range of physical parameters, residuals defined as $\epsilon = \frac{\ln {S_a(T)} - \ln {{\hat{S}}_a(T)}}{\text {std}\left( \ln {S_a(T)} - \ln {{\hat{S}}_a(T)}\right) }$(where ${\hat{S}}_a(T)$ is the reconstructed $S_a (T)$ spectra and $\text {std}\left( \ln {S_a(T)} - \ln {{\hat{S}}_a(T)}\right)$ is the standard deviation of the differences between the true and predicted) are analysed with respect to *M* and $R_{\textrm{rup}}$ at three representative *T* values: $T = 0.5,\text {s}, 1.0,\text {s}, 2.0,\text {s}$ (Fig. 3). This analysis is conducted on the natural–log scale commonly used in ground motion modelling, ensuring assumption of lognormality and direct comparability with standard residual diagnostics. This $\epsilon$ analysis allows for assessment of potential systematic biases in the model’s reconstructions and its ability to generalise across the ground motion parameter space.

The $\epsilon$ are generally centred around zero, with a dense concentration in the range $\epsilon \in [-3, 3]$, indicating an approximately normal distribution of residual errors and suggesting that the model captures the expected spectral amplitudes across the dataset without major bias. This indicates that the HVAE is not systematically over- or under-predicting across most of the dataset, and that $\epsilon$ errors primarily reflect sample-level variability. A closer inspection shows minor, physically interpretable, and statistically small trends at the upper end of *M*; these are expected under limited representation of very large events and reflect conservative regularisation rather than any systematic bias.

For example, at $T = 2.0,\text {s}$, the HVAE exhibits a slight, conservative underestimation for high-*M* events ($M > 6.5$) and corresponding mild overestimation for low *M* events ($M < 4.0$) (Fig. 3c). A similar observation can be made in Fig. 3a–b, but it is less prominent. This trend likely stems from attenuation in the training data distribution, which is imbalanced towards smaller earthquakes. Consequently, the model may default toward the conditional mean of spectral amplitudes, exhibiting a benign statistical shrinkage effect, particularly at longer *T* where the dynamic range of amplitudes is more pronounced. This is consistent with the physics of ground motion, where long-*T* spectral ordinates increase more steeply with *M* due to greater energy release and longer rupture durations^11,13^. In practice, this small monotonic tendency can be removed–if desired–via a lightweight post-hoc calibration (e.g., distance/magnitude-conditioned intercept or isotonic adjustment), without changing the core HVAE.

A similar tendency is observed across $R_{\textrm{rup}}$, where $\epsilon$ at $T = 0.5,\text {s}$ and $1.0,\text {s}$ suggest a slight, positive overestimation of acceleration for very large source-to-site distances ($R_{rup} > 150,\text {km}$) (Fig. 3d–e). These overestimations may reflect the challenges in capturing complex path effects, such as anelastic attenuation and regional crustal filtering, particularly in sparse observational regimes. Because motions at such large distances approach the noise floor, the absolute amplitudes are very small, so this bias is operationally insignificant for most engineering-demand use cases; it again could bias the model toward population-level averages in latent space. This observation is slightly less prominent for $T=2.0,\text {s}$ (Fig. 3f), although $|\epsilon |$ does appear to be slightly larger in general, likely reflecting the increased uncertainty in reconstruction for longer *T*. If required, a simple distance-conditioned correction term or weak attenuation prior can fully absorb this far-field effect.

These findings highlight the importance of considering the interaction between model architecture, data distribution, and the physics of ground motion in evaluating generative model performance. The observed asymmetries in $\epsilon$ are localised, small in magnitude, and consistent with well-understood data-coverage effects, and they do not materially affect the main conclusions or downstream tasks. For applications demanding additional tightening at these edges, trivial mitigations such as modest class-balancing or reweighting, weakly-informative priors on far-field attenuation, or a quantile-aware loss, can be applied without modifying the HVAE design. For seismic hazard applications, particularly those focused on rare but impactful large-*M* events, these edge-case behaviours mainly signal where additional data would yield the greatest marginal gains. It is also worth noting that similar or larger residual trends are commonly observed in conventional GMMs^11–13^, which report considerably higher prediction coefficients of variation across comparable *M*–$R_{\textrm{rup}}$ ranges, particularly at longer periods.

#### Latent group structure and geometric distortion

To assess the structural organisation induced by the hierarchical latent prior, both intra-group and inter-group distances are computed in the Poincaré ball $\mathbb {B}_c^d$ using the geodesic metric defined as Eq. 1, where $\textbf{z}_\textbf{x}, \textbf{z}_\textbf{y}$ are latent vectors of samples embedded in the Poincaré ball $\mathbb {B}_c^d$; $\Vert \cdot \Vert$ denotes the Euclidean norm; *c* is the negative curvature of the Poincaré ball $\mathbb {B}_c^d$; and $d_p^c(\cdot , \cdot )$ is the geodesic distance under hyperbolic geometry^43,47,48,59^. Intra-group distances quantify the cohesion among samples within an earthquake event, whereas inter-group distances measure the separation between event-level centroids. These centroids are computed using the Fréchet mean $\mu _{\textrm{F},g}$ of group *g*, defined as in Eq. 2, where $\varvec{\mu }_{\textrm{F},g}$ is the Fréchet mean (centroid) of group *g* in $\mathbb {B}_c^d$; and $G_g$ is the set of samples belonging to group *g*; and $\textbf{z}_\textbf{x}$ is the latent embedding of sample $\textbf{x}$^60^.

1 $$\begin{aligned} d_p^c(\textbf{z}_\textbf{x}, \textbf{z}_\textbf{y}) = \frac{1}{\sqrt{c}} \cosh ^{-1}\left( 1 + 2c \frac{\Vert \textbf{z}_\textbf{x} - \textbf{z}_\textbf{y}\Vert ^2}{(1 - c\Vert \textbf{z}_\textbf{x}\Vert ^2)(1 - c\Vert \textbf{z}_\textbf{y}\Vert ^2)}\right) , \quad \textbf{z}_\textbf{x}, \textbf{z}_\textbf{y} \in \mathbb {B}_c^d \end{aligned}$$

2 $$\begin{aligned} \varvec{\mu }_{\textrm{F},g} = \arg \min _{\textbf{z} \in \mathbb {B}_c^d} \sum _{\textbf{x} \in G_g} d_p^c(\textbf{z}, \textbf{z}_\textbf{x})^2 \end{aligned}$$

**Fig. 4:**
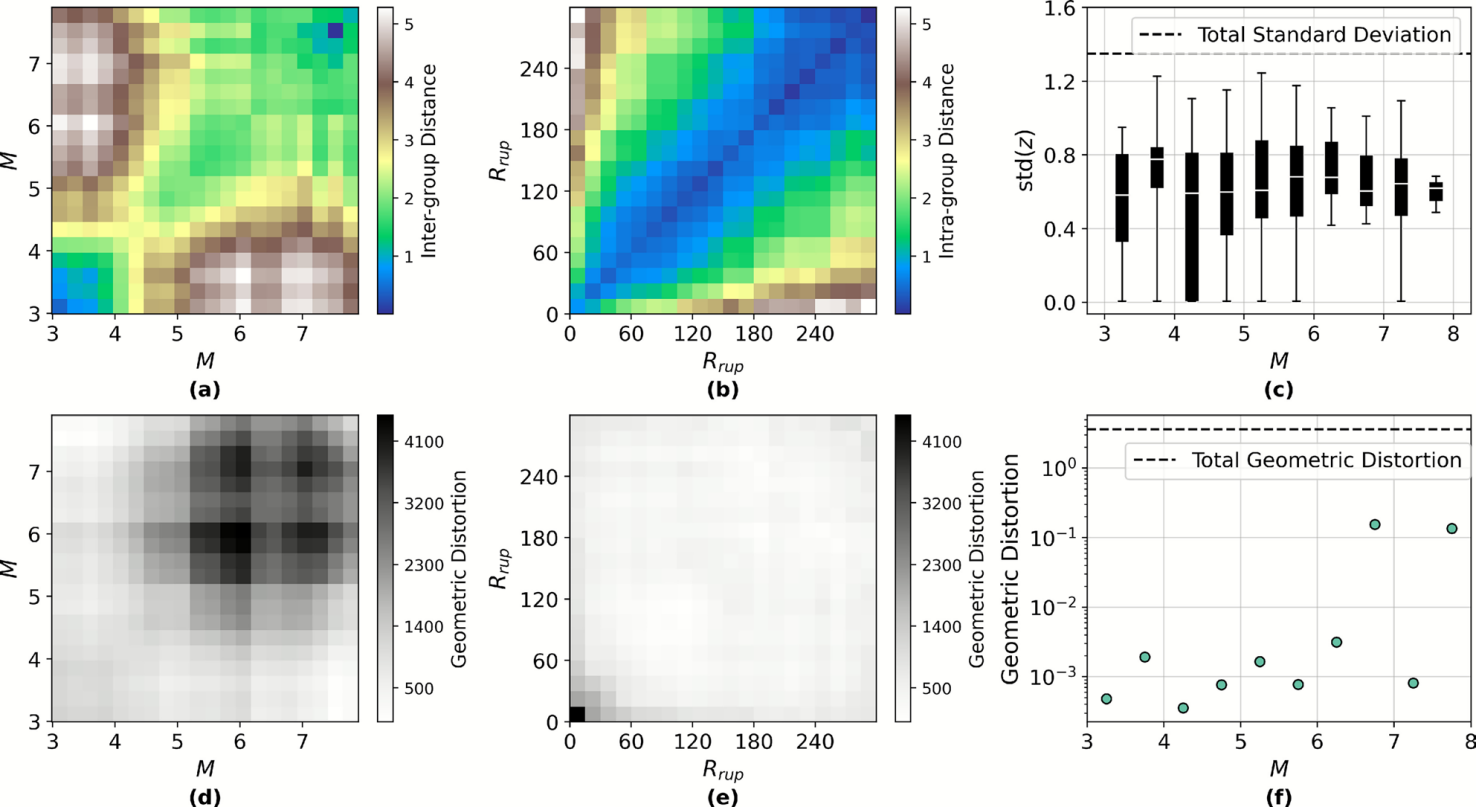
Inter-group distances and intra-group variance. (**a**) Heat map of distances between event centroids binned by magnitude *M*. (**b**) Heat map of distance between samples within groups binned by rupture distance $R_{\textrm{rup}}$. (**c**) Box plots of intra-group standard deviation for each event, binned by *M*; total standard deviation indicates the standard deviation for all samples. (**d**) Heat map of geometric distortion in the distance between event centroids, binned by *M*. (**e**) Heat map geometric distortion in the distance between samples within groups binned by rupture distance $R_{\textrm{rup}}$. (**f**) Scatter plot of geometric distortion in the average intra-group standard deviation for each event, binned by *M*; total geometric distortion indicates the distortion in the standard deviation for all samples.

Figure 4a demonstrates that events with disparate *M* (e.g., $M=3.5$ vs $M=7.0$) exhibit inter-group distances exceeding $d_p^c = 4.0$, confirming that the dispersion regularisation term effectively enforces event-level separability. Conversely, Fig. 4b reveals that intra-group distances, particularly between samples with a difference in $R_{\textrm{rup}}$ $< 60$ km, typically remain below $d_p^c = 1.0$, validating the efficacy of the cohesion term in preserving local proximity.

Latent spread within groups is further assessed using hyperbolic variance defined as in Eq. 3 where $\sigma _{\textrm{F},g}^2$ is the empirical hyperbolic variance of group *g*^60^. Figure 4c shows that the median intra-group standard deviation is approximately 0.6, significantly lower than the global standard deviation of all samples ($\sigma _{\text {total}} = 1.35$). This suggests tight clustering within events and confirms that the HVAE captures nested structure. An analysis of variance (ANOVA) test^61^ across *M* bins reveals statistically significant variation in intra-group spread ($p = 0.0250$), with the $M \in (4.0, 4.5]$ bin exhibiting the broadest interquartile range. A Mann–Whitney U test identifies this bin as significantly different from the others ($p = 0.00168$), likely due to a higher event density that concentrates embeddings near the origin.

3 $$\begin{aligned} \sigma _{\textrm{F},g}^2 = \frac{1}{|G_g|} \sum _{\textbf{x} \in G_g} d_p^c(\textbf{z}_\textbf{x}, \varvec{\mu }_{\textrm{F},g})^2 \end{aligned}$$

To evaluate curvature-induced distortion, the geometric distortion between each pair of latent points is defined as in Eq. 4, Where $\delta (\textbf{z}_\textbf{x}, \textbf{z}_\textbf{y})$ is the geometric distortion between $\textbf{z}_\textbf{x}$ and $\textbf{z}_\textbf{y}$^62^. Distortion maps in Fig. 4d–e show that inter-group distances are substantially inflated relative to their Euclidean counterparts, especially among high-*M* events–suggesting that those embeddings lie closer to the ball boundary where curvature magnifies distance. In contrast, intra-group distortions are minimal, implying that sample embeddings from nearby stations are concentrated near the origin.

4 $$\begin{aligned} \delta (\textbf{z}_\textbf{x}, \textbf{z}_\textbf{y}) = \frac{d_p^c(\textbf{z}_\textbf{x}, \textbf{z}_\textbf{y})}{\Vert \textbf{z}_\textbf{x} - \textbf{z}_\textbf{y}\Vert } \end{aligned}$$

Distortion in intra-group variance is also computed by comparing the hyperbolic and Euclidean spread as in Eq. 5, where $\text {Distortion}_{\sigma _{\textrm{F},g}}$ is the distortion in group *g*’s variance, $\sigma _{\textrm{F},g}^{\mathbb {B}}$ and $\sigma _{\textrm{F},g}^{\mathbb {E}}$ are the standard deviations in hyperbolic and Euclidean spaces, respectively^62^. As shown in Fig. 4f, curvature reduces intra-group variance while increasing the global variance, suggesting that Poincaré geometry enhances inter-group separation and intra-group compactness simultaneously. The overall geometric distortion of the latent space is summarised using the global distortion metric given in Eq. 6 where $\mathcal {D}_{\text {global}}$ is the average distortion across all latent vector pairs; *N* is the total number of latent vectors; and *Z* is the set of all latent vectors in the space^62^. This yields a value of $\mathcal {D}_{\text {global}} = 2.95$, indicating a global expansion of distances due to curvature.

5 $$\begin{aligned} \text {Distortion}_{\sigma _g} = \frac{\sigma _g^{\mathbb {B}}}{\sigma _g^{\mathbb {E}}} \end{aligned}$$

6 $$\begin{aligned} \mathcal {D}_{\text {global}} = \frac{1}{N^2} \sum _{(\textbf{z}_\textbf{x}, \textbf{z}_\textbf{y}) \in Z \times Z}{\delta (\textbf{z}_\textbf{x}, \textbf{z}_\textbf{y})} \end{aligned}$$

Collectively, these results confirm that the hierarchical regularisation and hyperbolic geometry enforce an interpretable and well-structured latent representation. The architecture effectively balances cohesion within seismic events and dispersion across events, consistent with the underlying geophysical hierarchy in earthquake ground motion.

#### Semantic continuity under latent interpolation

A key motivation for incorporating KL-divergence regularisation in VAEs is to enforce continuity and smoothness across the latent space, enabling semantically meaningful interpolations. In the HVAE, this smoothness is preserved within the hyperbolic latent space $\mathbb {B}_c^d$, where interpolation paths are naturally defined along geodesics due to the non-Euclidean geometry. To evaluate semantic consistency under latent traversal, geodesic interpolation is performed between two latent vectors $\textbf{z}_\textbf{x}$ and $\textbf{z}_\textbf{y}$ using the exponential and logarithmic maps on $\mathbb {B}_c^d$, expressed as Eq. 7 where $\gamma (t)$ denotes the interpolated latent representation at position *t* along the geodesic curve; $\text {exp}_{\textbf{z}_\textbf{x}}(\cdot )$ is the exponential map based at $\textbf{z}_\textbf{x}$; and $\text {log}_{\textbf{z}_\textbf{x}}(\cdot )$ is the logarithm map based at $\textbf{z}_\textbf{x}$^43,59^.

7 $$\begin{aligned} \gamma (t) = \text {exp}_{\textbf{z}_\textbf{x}}(t \cdot \text {log}_{\textbf{z}_\textbf{x}}(\textbf{z}_\textbf{y})), \quad t \in [0, 1] \end{aligned}$$

**Fig. 5:**
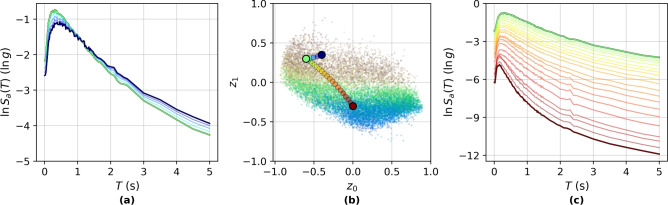
Smooth interpolation across the latent space. (**a**) Generated synthetic spectra from interpolation over a small distance, representing multiple recordings of the same event over different rupture distances. (**b**) Latent Space embeddings of generated spectra in (**a**) and (**c**), matched by colour. (**c**) Generated synthetic spectra from interpolation over a large distance, representing movement between recordings of different events.

Figure 5 presents the results of two representative interpolation scenarios. In the short-range case (Fig. 5a), interpolation is conducted between two samples belonging to the same seismic event with fixed *M*. The interpolated $S_a(T)$ exhibit smooth transitions in shape, with a systematic reduction in spectral amplitudes at short *T* ($T < 1.0$ s), particularly in *PGA*. This attenuation is consistent with increased source-to-site distance and reflects the attenuation of high-frequency content with distance^12^. The minor spectral broadening observed suggests that event-specific spectral features are preserved while accommodating intra-event variability.

In contrast, the long-range interpolation shown in Figure 5c spans between events of substantially different *M*. The generated $S_a(T)$ display a non-linear and monotonic decrease in amplitude across all *T*, suggesting a latent trajectory from a higher to a lower *M* event. Notably, a transient increase in spectral amplitude is observed around $T \approx 2.3$ s at an intermediate step of the geodesic, before flattening again at the target embedding. This transient peak is unlikely to emerge from simple averaging and highlights the HVAE’s ability to capture complex structural transitions in the ground motion manifold, possibly due to mode interactions or resonance phenomena typical of specific site-event combinations.

The latent interpolation trajectory is visualised in Figure 5b, where intermediate latent embeddings are colour-coded to match their generated $S_a(T)$. The colour continuity along the geodesic confirms smooth semantic transitions in latent space, with no evidence of discontinuities, degeneration, or collapse of learned structure.

These results demonstrate that the latent manifold learned by the HVAE enables continuous and physically meaningful synthesis of $S_a(T)$. The preservation of salient structural characteristics during interpolation, both within and across seismic events, confirms that the hierarchical and hyperbolic architecture captures geophysically grounded latent semantics. Furthermore, the curvature of the Poincaré ball appears to facilitate smooth navigation across varying IMs, thereby offering a controllable generative model for realistic ground motion $S_a(T)$ generation.

### Latent variable exploration

#### Latent dimensionality

**Fig. 6:**
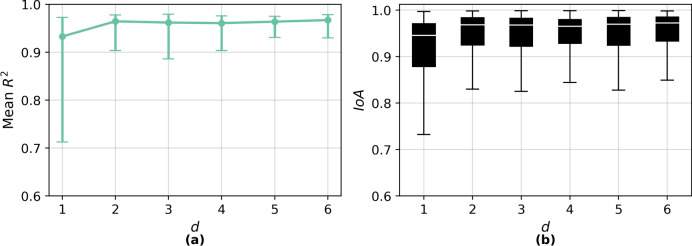
Reconstruction performance against latent dimensions *d*. (**a**) Mean coefficient of determination ($R^2$) with error bars are used to indicate the minimum and maximum $R^2$ values across the response spectrum periods. (**b**) Distribution of index of agreement (IoA) values across all samples.

The choice of latent dimensionality (*d*) plays a pivotal role in shaping both model performance and interpretability. While increasing $d$ often enhances the representational capacity of standard VAEs, this advantage does not linearly translate to performance gains in models constrained by non-Euclidean geometry^48,63^. Figure 6a shows the $R^2$ across *T* values, with error bars denoting the range from minimum to maximum values. Although the reconstruction performance improves significantly from $d = 1$ to $d = 2$, further increments in $d$ produce only marginal gains.

Similarly, the distribution of IoA values (Figure. 6b) suggests that $d > 2$ offer diminishing returns while increasing model complexity. From a geometric perspective, higher-*d* embeddings exacerbate curvature-induced distortion in the Poincaré ball $\mathbb {B}_c^d$^64^. The norm of a point $\textbf{z} \in \mathbb {B}_c^d$ is given by Eq 8 where $\textbf{z} = (z_0, z_1, \ldots , z_{d-1}) \in \mathbb {R}^d$ denotes a latent vector in the hyperbolic space and $\Vert \cdot \Vert _{\mathbb {B}_c^d}$ denotes the Euclidean norm constrained by the unit ball. As $d$ increases, the probability that a sampled latent vector approaches the unit boundary (i.e., $\Vert \textbf{z}\Vert \rightarrow \frac{1}{\sqrt{c}}$) also increases. This boundary region corresponds to regions of infinite hyperbolic distance and vanishing gradients, where optimisation becomes unstable. In practice, this occasionally manifests as negative KL-divergence during early training. To mitigate these effects while preserving interpretability, $d = 2$ is chosen for the final HVAE.

8 $$\begin{aligned} \Vert \textbf{z}\Vert _{\mathbb {B}_c^d} = \sqrt{z_0^2 + z_1^2 + \cdots + z_{d-1}^2} < \frac{1}{\sqrt{c}} \end{aligned}$$

#### Feature correlations and orthogonalisation effects

**Fig. 7:**
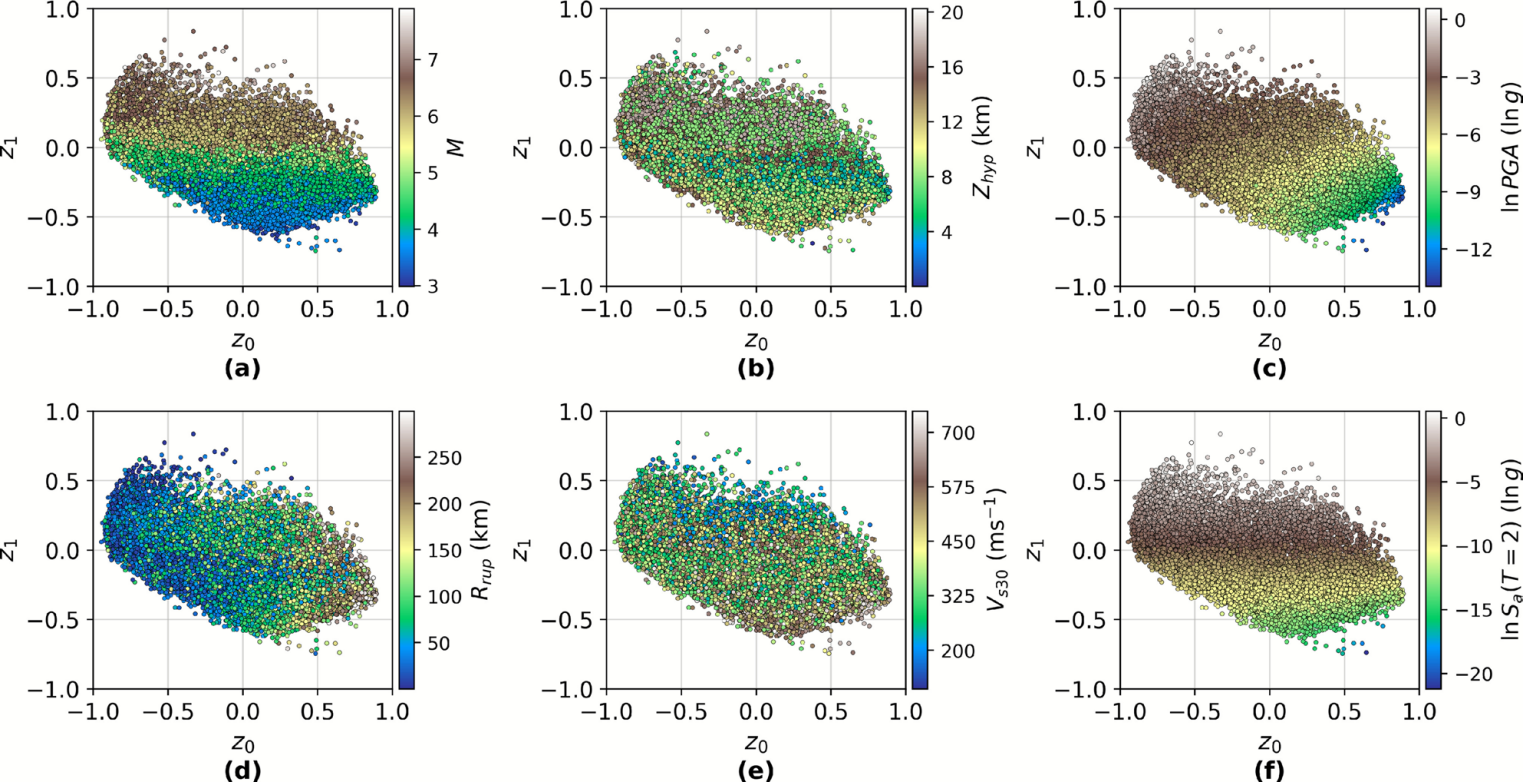
Latent space exploration. The latent space embeddings of all samples from both training and testing, colour-coded by (**a**) magnitude *M*, (**b**) hypocentre depth $Z_{\textrm{hyp}}$, (**c**) log-transformed peak ground acceleration $\ln {PGA}$, (**d**) rupture distance $R_{\textrm{rup}}$, (**e**) shear-wave velocity $V_{s30}$, and (**f**) log-transformed $S_a(T=2.0\text { s})$.

The HVAE incorporates both event-level features and sample-level features into the latent structure via regularisation loss terms (Eq. 14 and Eq. 15). To evaluate whether the latent space meaningfully encodes input variables, both global and intra-group correlations are computed between the latent variables $(z_0, z_1)$ and physical features including *M*, $Z_{\textrm{hyp}}$, $R_{\textrm{rup}}$, $V_{s30}$, and log-transformed *PGA*. Visual inspection of the 2D latent embeddings (Figure 7) reveals strong alignment along physically interpretable axes, particularly with $M$ and $\ln (PGA)$.

Based on the regularising loss terms introduced in Eq. 14 and Eq. 15, it is reassuring to observe that the latent space exhibits meaningful alignment with key physical features. In particular, a strong visual correlation is evident for *M* (Fig. 7a) and $R_{\textrm{rup}}$ (Fig. 7d), indicating that the learned latent variables effectively capture the most dominant sources of variability in $S_a(T)$. This outcome is consistent with prior work in ground motion prediction, where $M$ and $R_{rup}$ are known to have the largest influence on spectral amplitudes across a wide range of *T* values^41^. Their strong presence in the latent geometry suggests that the HVAE correctly leverages the imposed feature-level regularisation to anchor global structure in semantically relevant directions.

In contrast, the correlation of latent variables with $Z_{\textrm{hyp}}$ (Fig. 7b) and $V_{s30}$ (Fig. 7e) is weaker and less clearly delineated. This is likely due to two main factors. First, both $Z_{\textrm{hyp}}$ and $V_{s30}$ typically exert more localised and event- or station-specific influence on ground motion, which may be less prominent in the global latent geometry due to their lower variance across the dataset. Second, their effects on $S_a(T)$ are often non-linear and entangled with those of $M$ and $R_{rup}$, making them more difficult to disentangle through linear or weakly-regularised priors. The relative ambiguity observed in the latent projection for these features highlights the challenge of encoding finer-scale or second-order geophysical attributes within a low-dimensional latent manifold, particularly when the dominant spectral features are already captured by primary variables such as *M* and $R_{\textrm{rup}}$.

Quantitatively, $\rho$ computation^54^ (Table 1) confirms these observations. The strongest correlation is observed between $z_1$ and $M$ ($\rho = 0.876$) and between $z_0$ and $\ln (PGA)$ ($\rho = -0.875$), which aligns with the known coupling between *M* and peak ground motion. Moreover, $R_{rup}$ correlates positively with $z_0$ ($\rho = 0.563$ globally, $\rho = 0.555$ intra-group), indicating the horizontal latent axis encodes attenuation effects. Despite being included in the regularisation loss, $Z_{\textrm{hyp}}$ and $V_{s30}$ exhibit weaker global correlations (all $|\rho | < 0.31$), which is consistent with previous studies noting that these features have more diffuse influence on $S_a$ spectra.

**Table 1:** Correlation coefficients. $\rho$ computed between each feature and both latent variables. Intra-group refers to the mean $\rho$ computed per group across all groups.

Feature	Global $z_0$	Global $z_1$	Intra-group $z_0$	Intra-group $z_1$
Magnitude (*M*)	− 0.459	0.876	–	–
Hypocentre depth ($Z_{\textrm{hyp}}$)	− 0.267	− 0.212	–	–
Rupture distance ($R_{\textrm{rup}}$)	0.563	− 0.170	0.555	− 0.417
Shear-wave velocity ($V_{s30}$)	0.210	− 0.307	0.114	− 0.267
Log-transformed peak ground acceleration ($\ln {PGA}$)	− 0.875	0.809	− 0.745	0.479

The latent space forms an anisotropic embedding structure (Fig. 7), with a dominant gradient along $z_1$ capturing *M*-driven intensity variation, and a secondary, more dispersed structure along $z_0$ capturing $R_{\textrm{rup}}$-driven attenuation. Interestingly, the strong anti-correlation of $\ln (PGA)$ with $z_0$ and its positive correlation with $z_1$ may indicate a diagonal encoding (Fig. 7c) influenced by both *M* and $R_{\textrm{rup}}$, two parameters known to jointly modulate ground motion^41^. This is further reinforced by the global spread in $\ln S_a(T=2\,\textrm{s})$, shown in Fig. 7f, indicating the latent code captures broadband spectral effects.

The absence of strong $Z_{\textrm{hyp}}$ correlation across either latent dimension suggests that either: (i) its spectral signature is too subtle to disentangle with current supervision, or (ii) it is orthogonal to dominant sources of variance. This aligns with the hypothesis that $Z_{\textrm{hyp}}$ is partly absorbed in inter-group variations, and not readily decodable from the sample-wise $S_a(T)$.

To further assess the relationship between the latent variables and regularisation features, some sensitivity analysis is performed through use of random forest regressors^65^. The approach is to, for each feature, fit a random forest regressor with the latent variables as inputs to predict the feature in question. Following this, the regressor parameters is used to rank relative importance of the latent variables with respect to each feature. Furthermore, permutation importance is computed by randomly permutating the values of a singular latent dimension to remove collinearity between this dimension and the feature. The change in model error under this transformation is recorded, and the average of this change over multiple permutations is obtained.

The feature-importance results in Table 2 provide a quantitative assessment of how the HVAE latent dimensions encode the source, path, and site attributes introduced through the regularisation scheme. The relative-importance ranking shows a clear differentiation between the two latent variables: $z_{0}$ is more strongly associated with $R_{\textrm{rup}}$ and $V_{s30}$, whereas $z_{1}$ exhibits greater sensitivity to *M*. This aligns with the observed latent-space structure, where one axis primarily captures attenuation-driven variability and the other reflects event-level energy scaling.

**Table 2:** Feature importance as computed via random forest regressors of the latent variables to the regularisation features.

Feature	Relative importance		Mean permutation importance	
	$z_0$	$z_1$	$z_0$	$z_1$
Magnitude (*M*)	0.107	0.893	0.208	1.827
Hypocentre depth ($Z_{\textrm{hyp}}$)	0.510	0.490	1.126	1.002
Rupture distance ($R_{\textrm{rup}}$)	0.663	0.337	1.509	0.689
Shear-wave velocity ($V_{s30}$)	0.453	0.547	0.863	1.127

Permutation importance further reinforces these trends. Perturbing $z_{0}$ yields the largest degradation in predicting $R_{\textrm{rup}}$ (mean permutation importance $\approx 1.51$), while perturbing $z_{1}$ causes the greatest increase in error when predicting *M* ($\approx 1.83$). Both latent dimensions show comparable contributions to variability in $Z_{\textrm{hyp}}$ and $V_{s30}$, indicating that depth- and site-related intra-event effects are shared across the latent representation rather than isolated to a single axis.

#### Intra-group structure and group-level interpretability

**Fig. 8:**
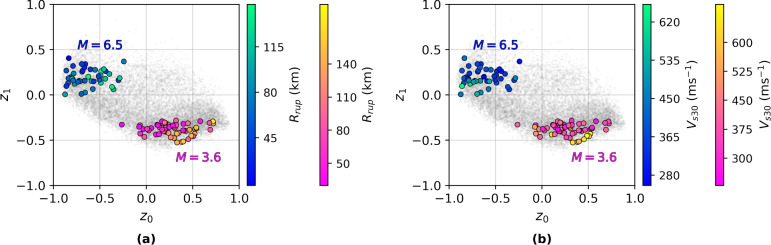
Latent space groupings. Latent embeddings from two events with magnitudes = 3.6 and 6.5, colour-coded by (**a**) rupture distance $R_{\textrm{rup}}$, (**b**) shear-wave velocity $V_{s30}$.

Given the hierarchical training structure, it is instructive to visualise latent encodings from individual seismic events. Figure 8 shows sample-wise latent codes from two distinct events with $M = 3.6$ and $M = 6.5$, coloured by $R_{\textrm{rup}}$ and $V_{s30}$. Despite global alignment of $M$ with $z_1$, within-group spread is primarily observed along $z_0$, especially with respect to $R_{\textrm{rup}}$ (Fig. 8)), affirming that intra-group variation aligns with distance and path effects. This is statistically supported by intra-group correlation in Table 1, where $R_{\textrm{rup}}$ exhibits strong positive correlation with $z_0$ ($\rho = 0.555$), while $V_{s30}$ (Fig. 8b) exhibits weaker correlation with $z_1$ ($\rho = -0.267$).

Such structured group-wise embeddings enable semantically meaningful interpolations and fine control over synthetic sample generation. The observed separation in Fig. 8 also indicates that the dispersion and cohesion terms in the loss effectively disentangle local (intra-event) variability from global (inter-event) trends.

### Downstream applications

The proposed HVAE framework supports multiple operational pathways by providing a compact, physically interpretable latent representation of earthquake $S_a$ spectra that can be queried, conditioned, and stochastically sampled. This section demonstrates two primary downstream applications: (i) integration into EEW workflows, where partial real-time observations are mapped to the latent space for rapid spectral inference, and (ii) feature conditioned forward simulation of site-specific ground motions, where latent samples are decoded into synthetic $S_a(T)$ and used to simulate acceleration time histories. Both applications leverage the hierarchical and hyperbolic structure of the HVAE latent space, enabling uncertainty-aware, physically consistent predictions that preserve inter-*T* dependencies and event–station variability.

#### Early warning via latent representation regression

The proposed HVAE architecture enables seamless integration within data-driven EEWS, serving as a generative decoder that reconstructs the full ground motion $S_a$ spectra from low-dimensional latent representations. This aligns with the motivation introduced in prior on-site EEWS frameworks^25,28^, where early-time features such as P-wave-derived IMs are leveraged for rapid estimation of $S_a(T)$. In contrast to traditional empirical GMM-based pipelines that rely on handcrafted parametric regressions, the present approach maps early IMs to a learned latent space that captures global and hierarchical structure in $S_a$ spectra.

**Fig. 9:**
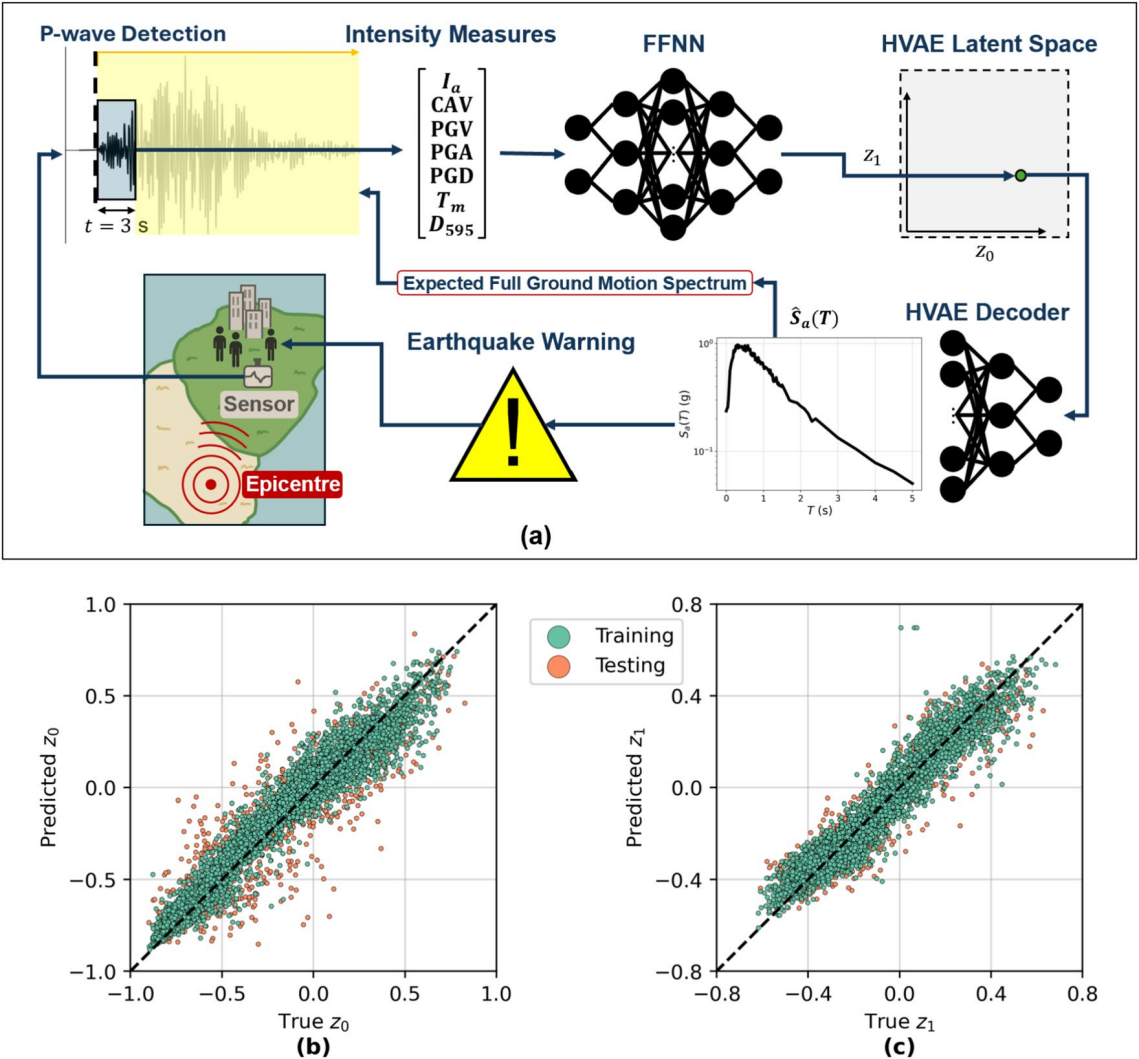
Earthquake early warning pipeline. (**a**) Earthquake early warning workflow: an on-site sensor monitors for any live ground motion. After P-wave detection, a three-second window of ground motion is used to compute a vector of intensity measures (IMs). The IM vector is fed to a pre-trained feed-forward neural network (FFNN) to estimate the corresponding point in the hierarchical variational autoencoder (HVAE) latent space. The estimated point is then decoded into the full response spectrum and used to conduct the final early warning. (**b**)-(**c**) True latent variables plotted against predicted latent variables from the FFNN, (**b**) $z_0$ ($R^2=0.907$ for all data) and (c) $z_1$ ($R^2=0.908$ for all data).

Figure 9a illustrates the full EEW pipeline. An on-site seismic sensor continuously monitors ground motion. Upon detection of a P-wave onset, the system triggers a processing window, analogous to the real-time detection strategy. Following P-wave detection, same as Fayaz and Galasso (2022)^28^, a three-second window of recorded acceleration is used to compute a set of IMs including Arias intensity ($I_a$), cumulative absolute velocity (*CAV*), peak ground velocity (*PGV*), *PGA*, peak ground displacement (*PGD*), mean *T* ($T_m$), and significant duration ($D_{595}$). In the proposed operational workflow, this three-second data acquisition phase is followed by less than one second of computation time for IM extraction, latent mapping, and spectrum reconstruction, ensuring that a full $S_a(T)$ prediction is available within approximately four seconds of P-wave onset. These features encapsulate signal amplitude, frequency content, and duration–key determinants of seismic demand–and have been previously shown to be predictive of spectral shape and amplitude^28^.

The extracted IM vector is fed into a fully connected FFNN, trained to map IMs to a low-dimensional point in the HVAE latent space $\textbf{z} = (z_0, z_1)$. This regression step replaces the direct GMM-style mapping with a learned latent representation that encodes hierarchical structure in $S_a(T)$. The predicted latent vector is subsequently passed to the HVAE decoder, which reconstructs the full multi-*T* response spectrum ${\hat{S}}_a(T)$ in real-time. This yields a full multi-*T* estimate of $S_a(T)$ within milliseconds of P-wave onset, without requiring information about source parameters or source-to-site geometry. This enables immediate estimation of shaking intensity across vibration periods relevant to engineering demand. The reconstructed $S_a$ spectra can be used in conjunction with application-specific thresholds–such as spectral displacement or intensity-based fragility curves–to determine whether to issue an early warning. This decision step is modular and can be tailored for on-site^28^ or regional EEW^25^ operations.

Training of the FFNN is conducted using an 80:20 train-test split, with model performance evaluated through $R^2$ values comparing predicted and true latent variables. As shown in Fig. 9b–c, the predicted ${\hat{z}}_0$ and ${\hat{z}}_1$ values show strong linear trend (i.e., purity) with the ground-truth latent variables, achieving $R^2 = 0.907$ and $R^2 = 0.908$ respectively across the full dataset. The training set achieved higher $R^2$ values (0.927 and 0.931 for $z_0$ and $z_1$ respectively), with only modest reduction on the test set ($R^2 = 0.831$ and 0.837), indicating reasonable generalisation and low risk of overfitting.

For comparison, a traditional GMM-based EEW pipeline follows a two-step procedure: (i) estimate source parameters such as *M* (and, in principle, $R_{\text {rup}}$) from early-time waveform features, and (ii) input these estimates into an empirical GMM to obtain $S_a(T)$. As discussed earlier and shown in Fig. 2b, even when the *true* *M* and distance values from the NGA-West2 database are provided to the GMM^12^, the resulting $S_a(T)$ predictions exhibit substantially lower fidelity (typically $R^2 \approx 0.45$–0.60). This represents the theoretical upper bound on GMM-based EEW performance, because no real-time estimator can provide source parameters with accuracy exceeding the use of true values. Any practical EEW implementation whether regression-based, ML-based, or DL-based, must introduce additional uncertainty through the real-time prediction of *M* and $R_{\text {rup}}$, further reducing reliability. Consequently, even with advanced *M* estimators or distance regression algorithms, GMM-based pipelines cannot surpass the performance ceiling illustrated in Fig. 2b.

In contrast, the HVAE-based approach bypasses explicit early-time estimation of *M* and $R_{\text {rup}}$ entirely. By inferring latent variables directly from early waveform intensity measures, the model avoids the compounded uncertainties inherent in two-step prediction pipelines and maintains coherent spectral predictions across *T*. This characteristic is particularly advantageous for EEW, where decisions rely on rapid and stable estimation of $S_a(T)$ rather than on intermediate source parameters.

Also unlike empirical or linear projection-based early warning methods, this approach captures non-linear interactions among IMs and exploits the latent geometry shaped by the HVAE. It also supports uncertainty-aware extensions (e.g., Bayesian FFNNs) and real-time deployment through lightweight computation. Furthermore, by mapping into a generative latent space, the framework can be extended to generate full $S_a(T)$ spectra for downstream use in structural fragility models, loss estimation, or ensemble forecasting under regional EEW architectures^25^.

Such low-latency spectrum reconstruction enables rapid, site-specific hazard estimation that can directly inform automated control actions, infrastructure shutdowns, and targeted evacuations. By delivering a full multi-period shaking estimate within seconds, the system supports time-critical decision making for operators, emergency managers, and lifeline services.

#### Ground motion time history simulation

A key advantage of the HVAE framework is that its latent space captures physically meaningful, feature-conditioned variability in spectral shapes, enabling stochastic ground motion simulation. Here, this capability is operationalised through a two-stage generative pipeline in which an auxiliary CVAE^66^ maps event- and site-level features directly to the HVAE latent space, followed by a physics-informed time-domain simulation algorithm.

The CVAE is conditioned on the set of predictor features $\{M, Z_{\textrm{hyp}}, R_{\textrm{rup}}, V_{s30}\}$. These features are passed through the CVAE encoder to produce the conditional latent variable $\textbf{u}$, parameterised by a mean vector $\varvec{\mu }_\textbf{u}$ and log-variance $\ln \varvec{\sigma }_\textbf{u}^2$, from which stochastic samples are drawn via the reparameterisation trick. The decoder then maps $\textbf{u}$ to the mean $\varvec{\mu }_\textbf{z}$ of the HVAE latent variables, $\textbf{z} = (z_0, z_1)$, which are paired with the log-variance $\ln \varvec{\sigma }_\textbf{z}^2$ to inject aleatoric variability into the spectra. The CVAE model performs reasonably well, achieving $R^2$ values of 0.812 and 0.901 with respect to the two latent variables of the HVAE, $z_0$ and $z_1$.

**Fig. 10:**
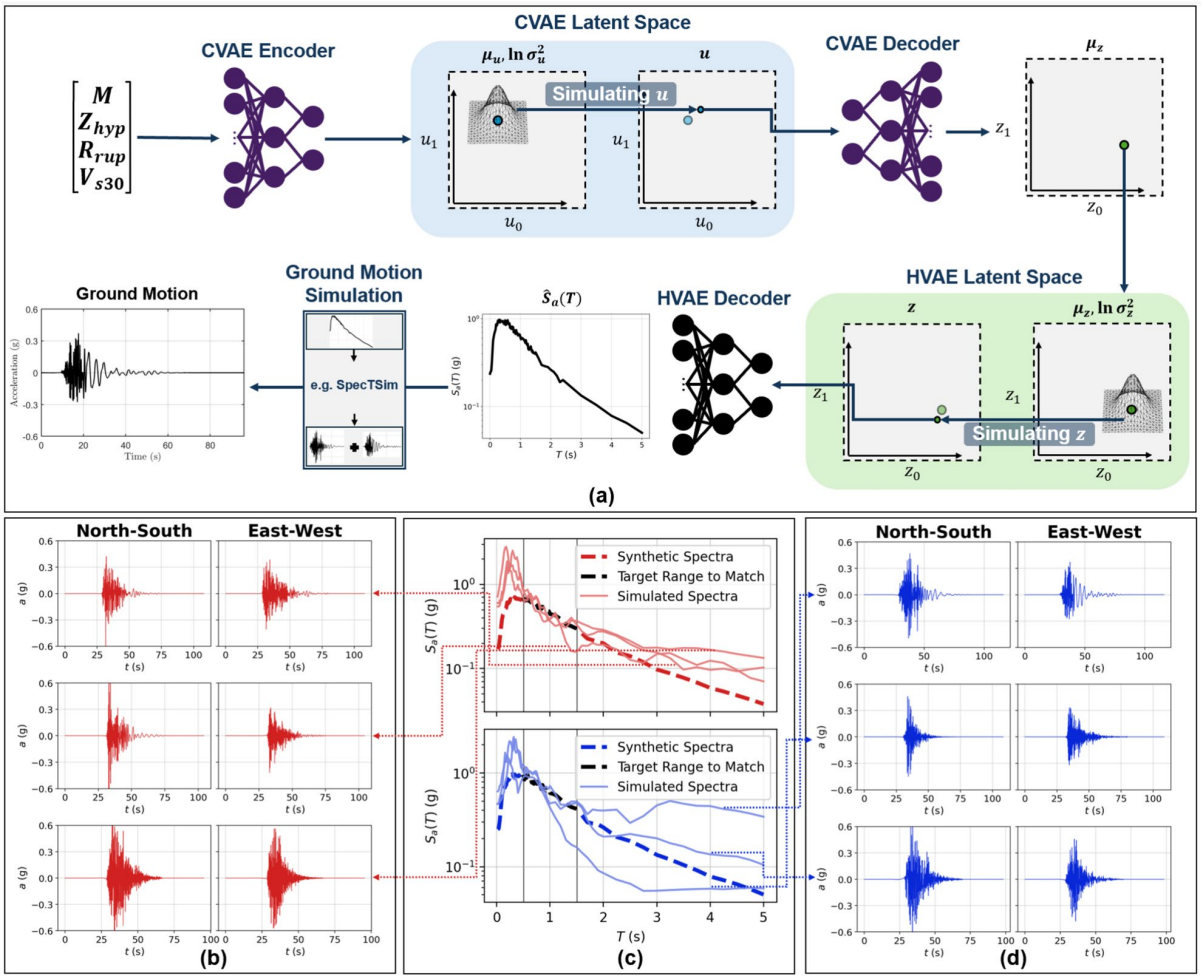
Ground motion simulation pipeline. (**a**) Ground motion simulation workflow: feature values are fed to the CVAE encoder to sample $\textbf{u}$ via reparameterisation. The sampled $\textbf{u}$ is then fed to the CVAE decoder to estimate $\varvec{\mu }_\textbf{z}$, which is paired with the HVAE’s log variance $\ln {\varvec{\sigma }_\textbf{z}^2}$ to sample $\textbf{z}$. The sampled $\textbf{z}$ is then fed to the HVAE decoder to generate a corresponding synthetic response spectrum ${\hat{S}}_a(T)$ which is then used as a target spectrum to synthesise ground motion time histories via algorithms like SpecTSim. (**b**) Three simulated ground motion time histories in the two orthogonal horizontal directions. (**c**)-top The target synthetic response spectrum used to simulate the ground motions in (**b**), overlayed by the spectra of the simulated ground motions. (**c**)-bottom The target synthetic response spectrum used to simulate the ground motions in (**d**), overlayed by the spectra of the simulated ground motions. (**d**) Three simulated ground motions split into orthogonal horizontal directions.

Once sampled, $\textbf{z}$ is decoded by the pre-trained HVAE decoder to yield a synthetic target response spectrum ${\hat{S}}_a(T)$. The choice of generating ${\hat{S}}_a(T)$ in the HVAE space, rather than directly in the CVAE output, ensures that hierarchical structure and hyperbolic geometry regularisation, both critical for capturing inter-event dispersion and intra-event clustering, are retained in the generated $S_a(T)$ spectra. This is employed to generate multiple $S_{a}(T)$ spectra realisation for the maximum considered earthquake (MCE) level scenario (return period = 2500 years) for downtown Los Angeles, USA, with *M* = 6.91, $R_{rup}$ = 11.04 km, and $V_{s30}$ = 360 m/s.

The resulting ${\hat{S}}_a(T)$ (RotD50 spectra) is used as targets to simulate site-based acceleration time histories^67,68^ using the SpecTSim algorithm^69^, which performs an efficient iterative spectral matching procedure that enforces simulated ground motion time histories to match ${\hat{S}}_a$ within a target range of *T* while maintaining realistic non-stationary and phasing characteristics. Specifically, the algorithm produces physically consistent pairs of orthogonal accelerograms, generating the major–intermediate directions for non-pulse motions or the maximum-pulse direction for pulse-like records. This method achieves high computational efficiency while avoiding the non-physical artefacts that can arise from naive scaling. This approach yields time-history sets that are consistent with engineering demand modelling requirements.

Figure 10a illustrates the complete workflow, from feature-conditioned CVAE sampling to HVAE decoding and time-history synthesis. Fig. 10b and d show examples of simulated ground motions, while Figure 10c (top) and (bottom) demonstrate the agreement between the synthetic target $S_a(T)$ spectra and the $S_a(T)$ spectra of the simulated time histories. In this case, the ground motions are simulated conditioned on the target *T* of 0.5 s to 1.5 s. The close alignment across the target *T* range confirms the spectral fidelity of the approach, with deviations primarily observed at short *T* ($T < 0.2$ s) due to the inherent amplification of high-frequency noise during spectral matching an issue also noted in^69^. A similar operational process was followed for benchmarking, in which 10 ground motions were simulated for 10 distinct target spectra, with an average runtime of under one minute per time history generation (including spectral matching) on standard workstation hardware.

Critically, the stochasticity in both the CVAE and HVAE latent sampling ensures that multiple, statistically distinct ground motions can be simulated for the same feature set, thereby enabling hazard-consistent scenario generation. This contrasts with deterministic GMMs, which yield a single mean prediction and cannot explore the distributional tails relevant for performance-based seismic analysis. Moreover, because the HVAE latent variables are interpretable with respect to physical predictors, the framework provides an explicit mechanism to explore how variations in *M*, $Z_{\textrm{hyp}}$, $R_{\textrm{rup}}$, and $V_{s30}$ map to changes in spectral shape and, ultimately, time-history characteristics.

The ability to generate large ensembles of realistic, site-specific ground motions conditioned on scenario parameters provides a powerful tool for PSHA, performance-based structural design, and portfolio-level risk assessment. These simulations can capture hazard-consistent variability, supporting both long-term resilience planning and regulatory compliance. Although this pipeline is not for applications directly within EEW, it is interesting to note that the computational time of the ML components at inference is negligible at approximately $43\pm 15\text { ms}$. On the other hand SpecTSim algorithm takes approximately $76\pm 29\text { s}$ per simulation, giving a total computational time of approximately $76.043\pm 29.015\text { s}$ per ground motion simulation. It should also be noted that the SpecTSim algorithm is currently written in Matlab and, with better packaging in C/C++ or similar, the computational time can certainly be reduced further (Andrews [2012] quoted a 500 times speed increase with C++ compared to Matlab^70^).

This modular design is generalisable: the CVAE–HVAE–SpecTSim chain can be embedded into PSHA and other ground motion simulation workflows to produce ensembles of site-specific motions conditioned on scenario parameters, or integrated with real-time EEW systems to rapidly simulate plausible shaking histories for operational response planning.

## Conclusion

This study presents an HVAE formulated in a hyperbolic latent space for the generative modelling of earthquake $S_a(T)$. The architecture encodes recordings in a two-tier latent structure that separates inter-event from intra-event variability, regularised by physically meaningful features and embedded in a Poincaré ball to exploit the efficiency of hyperbolic geometry for hierarchical representation. Training on a curated subset of NGA-West2 demonstrates that the HVAE preserves cross *T* correlation structure, achieves a mean $R^2$ of 0.961 across all *T* values, and maintains semantic continuity under geodesic interpolation. The only performance limitation lies in a minor bias towards small-to-moderate *M* events, as evidenced with the IoA metric. Future work should be done to further address this from a generative modelling perspective. However, it should be noted that there was no bias observed with respect to EEW performance, demonstrating that the practical impact is minimal.

From an ML perspective, the framework demonstrates how domain-informed hierarchical priors, feature-aligned regularisation, and non-Euclidean geometry can be combined to produce a generative model whose latent manifold aligns with physically meaningful axes (e.g., *M* and $R_{\textrm{rup}}$), while retaining smoothness for geodesic interpolation. This enables both semantic control and stochastic diversity in forward sampling. The modular design further supports downstream integration: an FFNN→HVAE coupling for rapid early-warning inference from partial P-wave IMs, and a CVAE-HVAE-SpecTSim chain for site-specific, hazard-consistent ground motion simulation. In both cases, the use of a shared, interpretable latent space ensures that predictive and generative tasks are geometrically and physically consistent.

From a seismological perspective, the HVAE effectively disentangles *M*-driven intensity scaling from distance-driven attenuation, reflecting established ground motion scaling theory. The hierarchical and geometric design captures nested variability between stations and events, which is essential for simulating statistically distinct yet physically plausible motions for PSHA, EEW, performance-based design, and operational earthquake forecasting. The stochasticity in both the CVAE and HVAE latent sampling allows exploration of distributional tails, addressing a limitation of deterministic models that produce only mean predictions. The demonstrated ML-based application to EEW is highly computationally efficient, a real-time pipeline delivering warnings in approximately $33\pm 7\text { ms}$ of a $3\text { s}$ window following P-wave detection (based on 1,000 test runs of a single sample). This implies, a warning can be issued within approximately $3.03 \text { s}$ of P-wave detection, enabling a rapid response time.

The results confirm that integrating hierarchical priors, non-Euclidean latent spaces, and domain-aligned regularisation yields a generative framework that is both interpretable and uncertainty-aware. This work demonstrates that geometric DL principles can be operationalised for high-impact seismological applications, bridging real-time tasks such as early warning with long-term hazard modelling. The proposed model provides a rigorous proof of concept and, similar to GMMs, can be trained independently for different regions to obtain localised latent spaces and fine-tuned model performance. Future work should explore adaptive prior variance to modulate spectral diversity, deeper hierarchical tiers to capture additional physical groupings (e.g., fault type, regionalisation), and hybrid physics–ML training regimes to improve extrapolation in sparse data regimes. Moreover, the framework’s generality suggests applicability beyond seismology to other hazard-modelling domains where structured variability, uncertainty quantification, and generative capability are equally critical. In bridging geometric DL and earthquake engineering, this study provides a foundation for interpretable, uncertainty-aware generative models that can support both real-time early warning and long-term seismic hazard mitigation.

## Methods

This study develops and evaluates a hierarchical generative modelling framework for earthquake $S_a(T)$, formulated as a VAE with event–station latent priors embedded in a hyperbolic manifold. The architecture is designed to jointly capture inter-event and intra-event variability by imposing a two-level latent structure regularised by physically meaningful features. The Methods section details the dataset curation and preprocessing, the formulation of the HVAE loss functions, the integration of hyperbolic geometry into the latent representation, and the configuration of downstream neural modules for early warning and ground motion simulation. All methodological components are implemented to maintain physical plausibility while enabling uncertainty-aware generative capability.

### Dataset

**Fig. 11:**
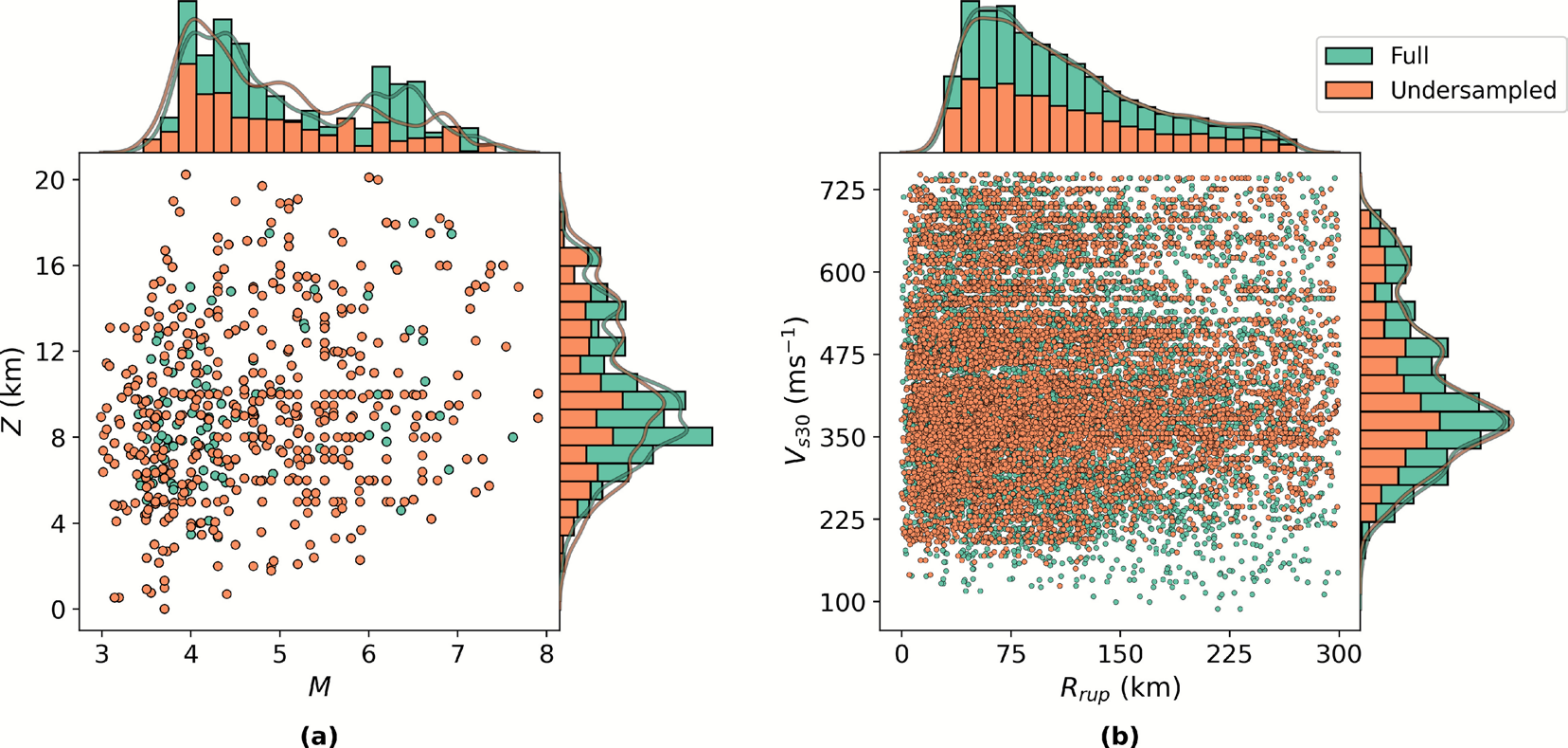
Data undersampling. The distribution of samples in the dataset by (**a**) magnitude *M* and hypocentre depth $Z_{\textrm{hyp}}$, and (**b**) rupture distance $R_{\textrm{rup}}$ and shear-wave velocity $V_{s30}$, overlayed with an 50% resampled subset using KDE undersampling.

The training corpus is derived from the NGA-West 2 ground motion database^71^, comprising 21,540 recordings from 600 earthquakes across 3,039 seismic stations. Each sample contains: (i) event-level features (e.g., *M*, $Z_{\textrm{hyp}}$), (ii) sample-level features (e.g., $R_{\textrm{rup}}$, $V_{s30}$), and (iii) $S_{a}(T)$ response function. Since $S_{a}(T)$ is a continuous variable in *T*, it is discretised over 91 periods in the range 0.0 to 5.0 s. All spectral ordinates are log-transformed to stabilise variance and reduce skewness^72^.

Data quality control removes outliers by filtering events with $Z_{\textrm{hyp}} \ge 20\,\textrm{km}$, and recordings with $R_{\textrm{rup}} \ge 300\,\textrm{km}$ or $V_{s30} \ge 750\,\mathrm {ms^{-1}}$, resulting in 18,493 valid samples from 573 events. To mitigate multivariate continuous class imbalance, notably bimodality in *M*, the study applies kernel density estimate (KDE) undersampling in the joint feature space $(M, Z_{\textrm{hyp}}, R_{\textrm{rup}}, V_{s30})$. This resampling is weighted inversely to the estimated density, yielding a balanced representation of medium-*M* events and preserving the distribution tails. An 80% KDE undersample reduces the final modelling set to 14,908 samples from 553 unique events, ensuring better latent space regularisation and improved generalisation.

Figure 11 illustrates the effect of KDE undersampling. Panel (a) shows the distribution of *M* versus $Z_{\textrm{hyp}}$, revealing the initial bimodality in *M* and the reduction of overrepresented low- and high-*M* clusters after undersampling. Panel (b) presents $R_{\textrm{rup}}$ versus $V_{s30}$, highlighting the preservation of distribution tails while reducing density in overrepresented regions of the feature space. The marginal histograms confirm that KDE undersampling selectively reduces densely populated regions without imposing hard bin thresholds, thus retaining the physical diversity of the dataset while improving class balance in continuous variables.

### Neural network training

#### Variational autoencoder

The core generative backbone is implemented as a VAE^37^, extending the formulation of Fayaz et al.^25^ to incorporate hierarchical structure and physics-informed regularisation. A VAE consists of two coupled neural networks: an encoder, parameterised by $\phi$, which maps input $S_a(T)$ to a low-dimensional latent representation, and a decoder, parameterised by $\theta$, which reconstructs $S_a(T)$ from this latent code (${\hat{S}}_a(T)$). The model is trained by maximising the evidence lower bound (ELBO) on the marginal log-likelihood of the observed data, balancing spectral reconstruction accuracy with statistical regularisation of the latent distribution^37^.

Given a batch *B* of samples $\textbf{x}$, the base VAE loss comprises: (i) a reconstruction term, $L_{\textrm{recon}}$, defined as the mean-squared error between $\textbf{x}$ and its reconstruction $\mathbf {{\hat{x}}}$; and (ii) a KL-divergence term, $L_{\textrm{KL}}$, which penalises deviation of the encoder’s posterior distribution $\mathcal {N}(\varvec{\mu }_\phi (\textbf{x}), \varvec{\sigma }^2_\phi (\textbf{x}))$ from the standard normal prior $\mathcal {N}(\textbf{0}, \textbf{I})$. This yields Eq. 9, where *d* is the latent dimensionality with *i* used as an index and $\varvec{\mu }_\phi (\textbf{x})$, $\ln {\varvec{\sigma }^2_\phi (\textbf{x})}$ are the encoder outputs:

9 $$\begin{aligned} \mathcal {L}_{\textrm{VAE}}(\theta , \phi ; B) = \frac{1}{|B|}\sum _{\textbf{x} \in B}{ \left( \underbrace{ \Vert \textbf{x} - \mathbf {{\hat{x}}}\Vert ^2}_{L_{\textrm{recon}}} + \underbrace{ \frac{1}{2} \sum _{i=0}^{d-1} \left[ \varvec{\sigma }^2_\phi (\textbf{x})_i + \varvec{\mu }_\phi (\textbf{x})_i^2 - \ln \varvec{\sigma }^2_\phi (\textbf{x})_i - 1 \right] }_{L_{\textrm{KL}}} \right) } \end{aligned}$$

To embed physically meaningful structure into the latent space, an additional feature-anchored regularisation term $L_{\textrm{reg}}$ is introduced, encouraging proximity in latent space for samples that are similar with respect to selected physical features (e.g., *M*, $Z_{\textrm{hyp}}$, $R_{\textrm{rup}}$, $V_{s30}$). This is expressed in Eq. 10 where $\tau _f$ is the dataset-wide standard deviation of feature *f*, $f_\textbf{x}$ is its value for sample $\textbf{x}$, and *F* denotes the set of selected features. This term implements a feature-dependent partial pooling, acting as a soft constraint that aligns latent topology with known seismological dependencies.

10 $$\begin{aligned} L_{\textrm{reg}} = \frac{1}{|B|^2} \sum _{(\textbf{x}, \textbf{y}) \in B \times B} \exp \left( -\sum _{f \in F} \frac{\Vert f_\textbf{x} - f_\textbf{y}\Vert ^2}{\tau _f} \right) \cdot \Vert \textbf{z}_\textbf{x} - \textbf{z}_\textbf{y} \Vert ^2 \end{aligned}$$

Eq. 11 presents the final batch-wise objective that combines these components with scalar weights $W_{\textrm{recon}}$, $W_{\textrm{KL}}$, and $W_{\textrm{reg}}$ to control their relative influence. This formulation not only enforces statistical coherence and reconstruction fidelity but also integrates domain-specific inductive biases directly into the optimisation, ensuring that the learned latent variables are both generatively expressive and seismologically interpretable.

11 $$\begin{aligned} \mathcal {L}(\theta , \phi ; B, F) = W_{\textrm{recon}} L_{\textrm{recon}} + W_{\textrm{KL}} L_{\textrm{KL}} + W_{\textrm{reg}} L_{\textrm{reg}} \end{aligned}$$

#### Hierarchical embeddings

To capture inter-sample dependencies arising from shared earthquake events (groups), the latent prior is extended to a hierarchical formulation in which each group *g* is assigned its own Gaussian prior distribution^54^. Under this setting, the latent embedding *z* for a sample from group *g* is drawn from $\textbf{z} \sim \mathcal {N}(\varvec{\mu }_g, \varvec{\sigma }_g^2)$, where the group mean $\varvec{\mu }_g$ itself follows a hyper-prior $\varvec{\mu }_g \sim \mathcal {N}(\textbf{0}, \textbf{I})$. In the general case, intra-group variances could be drawn from a half-normal distribution, but for tractability, $\varvec{\sigma }_g$ is fixed as a scalar constant across dimensions ($\sigma _g$). This yields a two-tiered latent structure: globally, all embeddings conform to a standard normal distribution; locally, within an event, samples are clustered in a smaller, event-specific Gaussian.

This structure encodes spatial and event-based correlation in the latent space: sampling a single group mean $\varvec{\mu }_g$ generates a canonical latent representation for the event, while perturbations around this mean produce $S_a$ spectra reflecting multiple recordings from that event. The formulation naturally supports event-conditioned generation and strengthens the interpretability of latent clusters in seismological terms.

The hierarchical prior modifies the standard KL-divergence loss $L_{\textrm{KL}}$ to measure divergence from the event-specific prior rather than a global prior, and introduces an additional hyper-prior KL term $L_{\textrm{KL,hyp}}$ to regularise the distribution of group means. This is expressed as Eqs. 12 and  13 where *G* is the set of group indices; $G_g$ is the set of samples for group *g*; $\varvec{\mu }_G$ and $\varvec{\sigma }_G^2$ are the empirical mean and variance of $\{\varvec{\mu }_g\}$.

12 $$\begin{aligned} L_{\textrm{KL}} = \frac{1}{|B|} \sum _{g \in G} \sum _{\textbf{x} \in G_g} \frac{1}{2} \sum _{i=0}^{d-1} \left[ \log \left( \frac{\sigma _g^2}{\varvec{\sigma }^2_\phi (\textbf{x})_i} \right) + \frac{\varvec{\sigma }^2_\phi (\textbf{x})_i}{\sigma _g^2} + \frac{(\varvec{\mu }_\phi (\textbf{x})_i - \varvec{\mu }_{g, i})^2}{\sigma _g^2} - 1 \right] \end{aligned}$$

13 $$\begin{aligned} L_{\textrm{KL,hyp}} = \frac{1}{2} \sum _{i=0}^{d-1} \left[ \varvec{\sigma }_{G, i}^2 + \varvec{\mu }_{G, i}^2 - \log (\varvec{\sigma }_{G, i}^2) - 1 \right] \end{aligned}$$

Since the model explicitly groups samples by event, global regularisation using sample-level features (e.g., $R_{\textrm{rup}}$) is inappropriate, as these vary substantially within an event. Instead, regularisation is applied at two levels: (i) inter-group regularisation, using event-level features to constrain proximity between group means; and (ii) intra-group regularisation, using sample-level features to control dispersion within an event.

For the group level, a hyper-regularisation term $L_{\textrm{reg,hyp}}$ combines a cohesion component (pulling similar group means together) and a dispersion component (ensuring minimum separation between dissimilar groups). This is expressed in Eq. 14 where *s* enforces a base separation; $F_{\textrm{hyp}}$ is the set of hyper-regularisation features (e.g. *M*, $Z_{\textrm{hyp}}$); $f_g$, $f_h$ are the values of feature *f* for groups *g*, *h* respectively ; and $\varvec{\mu }_g$, $\varvec{\mu }_h$ are the prior means for groups *g*, *h* respectively;.

14 $$\begin{aligned} L_{\textrm{reg,hyp}} = \frac{1}{|G|^2} \sum _{(g,h) \in G \times G} \left[ e^{-\sum _{f \in F_{\textrm{hyp}}} \frac{\Vert f_g - f_h\Vert ^2}{\tau _f}} \cdot \Vert \varvec{\mu }_g - \varvec{\mu }_h\Vert ^2 + e^{-\Vert \varvec{\mu }_g - \varvec{\mu }_h\Vert ^2} \cdot \left( \sum _{f \in F_{\textrm{hyp}}} \frac{\Vert f_g - f_h\Vert ^2}{\tau _f} + s\right) \right] \end{aligned}$$

At the intra-group level, regularisation $L_{\textrm{reg}}$ is similarly formulated but applied only to pairs of samples $(\textbf{x},\textbf{y})$ within the same group in Eq. 15.

15 $$\begin{aligned} L_{\textrm{reg}} = \frac{1}{|B|^2} \sum _{g \in G} \sum _{(\textbf{x},\textbf{y}) \in G_g \times G_g} \left[ e^{-\sum _{f \in F} \frac{\Vert f_\textbf{x} - f_\textbf{y}\Vert ^2}{\tau _f}} \cdot \Vert \textbf{z}_\textbf{x} - \textbf{z}_\textbf{y}\Vert ^2 + e^{-\Vert \textbf{z}_\textbf{x} - \textbf{z}_\textbf{y}\Vert ^2} \cdot \sum _{f \in F} \frac{\Vert f_\textbf{x} - f_\textbf{y}\Vert ^2}{\tau _f} \right] \end{aligned}$$

The full batch-wise objective thus incorporates reconstruction, KL-divergence at both sample and group levels, and corresponding regularisation terms, as expressed in Eq. 16. This hierarchical embedding framework enforces multi-scale latent structure, aligning global topology with event-level physics while preserving intra-event variability critical for ground motion representation.

16 $$\begin{aligned} \mathcal {L}(\phi , \theta , \{\mu _g\}; B, F, F_{\textrm{hyp}}, \textbf{W}, G) = \textbf{W}^\top \textbf{L},\quad \quad \textbf{W} = \begin{bmatrix} W_{\textrm{recon}} \\ W_{\textrm{KL}} \\ W_{\textrm{reg}} \\ W_{\textrm{KL,hyp}} \\ W_{\textrm{reg,hyp}} \end{bmatrix}, \quad \textbf{L} = \begin{bmatrix} L_{\textrm{recon}} \\ L_{\textrm{KL}} \\ L_{\textrm{reg}} \\ L_{\textrm{KL,hyp}} \\ L_{\textrm{reg,hyp}} \end{bmatrix} \end{aligned}$$

#### Hyperbolic geometry

The HVAE is tasked with embedding nearly 15,000 ground-motion recordings from 553 earthquake events, organised in a two-tier hierarchy: individual recordings (samples) are nested within seismic events (groups). This structure requires the latent space to simultaneously preserve global inter-event topology and local intra-event variation. Standard Euclidean embeddings struggle to allocate representational capacity efficiently in such hierarchical settings. Hyperbolic geometry, in particular the Poincaré ball model, provides a mathematically natural alternative. With constant negative curvature, the Poincaré ball embeds tree-like or hierarchical structures with low distortion, allowing exponentially increasing representational space near its boundary^43,47,48,59^.

Formally, the *d*-dimensional Poincaré ball of curvature $-c$ is defined as Eqs. 17 and  18 where $\textbf{v}, \mathbf {{\tilde{v}}} \in \mathbb {B}_c^d$ and $\Vert \cdot \Vert$ denotes the Euclidean norm. Distances increase rapidly near the ball’s edge, allowing fine-grained separation between unrelated events while maintaining compactness for related ones.

17 $$\begin{aligned} \mathbb {B}_c^d = \left\{ \textbf{v} \in \mathbb {R}^d: \Vert \textbf{v}\Vert < \frac{1}{\sqrt{c}} \right\} \end{aligned}$$

18 $$\begin{aligned} d_p^c(\textbf{v}, \mathbf {{\tilde{v}}}) = \frac{1}{\sqrt{c}} \cosh ^{-1} \left( 1 + \frac{2c \Vert \textbf{v} - \mathbf {{\tilde{v}}}\Vert ^2}{(1 - c\Vert \textbf{v}\Vert ^2)(1 - c\Vert \mathbf {{\tilde{v}}}\Vert ^2)} \right) \end{aligned}$$

To perform variational inference in this geometry, probability distributions in the latent space must respect hyperbolic curvature. This study adopts the *wrapped normal* distribution (parameterised by mean and variance $\varvec{\mu }$ and $\varvec{\sigma }$)^48,63^, which is constructed by sampling from a Gaussian in the tangent space $T_{\varvec{\mu }}\mathbb {B}_c^d$, scaling by the conformal factor $\lambda _{\varvec{\mu }}^c = \frac{2}{1 - c\Vert \varvec{\mu }\Vert ^2}$, and mapping to the manifold via the exponential map (Eq. 19) where $\oplus _c$ denotes Möbius addition^48,59^. The inverse mapping is given by the logarithmic map $\log _{\varvec{\mu }}^c(\textbf{z})$, which returns the tangent vector from $\varvec{\mu }$ to $\textbf{z}$^48,59^. The wrapped normal probability density function for some $\textbf{z}\in \mathbb {B}_c^d$ incorporates a curvature-dependent change of variables (Eq. 20)^48^.

19 $$\begin{aligned} \exp _{\varvec{\mu }}^c(\textbf{v}) = \varvec{\mu } \oplus _c \left( \tanh \left( \sqrt{c} \frac{\lambda _{\varvec{\mu }}^c \Vert \textbf{v}\Vert }{2} \right) \frac{\textbf{v}}{\sqrt{c} \Vert \textbf{v}\Vert } \right) \end{aligned}$$

20 $$\begin{aligned} \mathcal{W}\mathcal{N}_c(\textbf{z}|\varvec{\mu }, \varvec{\sigma }) = \mathcal {N}\left( \lambda _{\varvec{\mu }}^c \log _{\varvec{\mu }}^c(\textbf{z}) \,\Big |\, \textbf{0}, \varvec{\sigma }\right) \left( \frac{\sqrt{c} \, d_p^c(\varvec{\mu }, \textbf{z})}{\sinh \left( \sqrt{c} \, d_p^c(\varvec{\mu }, \textbf{z}) \right) } \right) ^{d-1} \end{aligned}$$

In the HVAE, both the prior and the posterior distributions are modelled as wrapped normals. The KL-divergence between these distributions is approximated by evaluating the difference of their log-densities at a single posterior sample^63^. While this is a coarse approximation, the small posterior variances and large dataset size make it effective in practice.

Architecturally, the encoder and decoder remain Euclidean for computational tractability, with the latent layer operating in $\mathbb {B}_c^d$. In the encoder, the log-variance $\ln \varvec{\sigma }_\phi ^2(\textbf{x})$ is used directly in the tangent space, while the mean $\varvec{\mu }_\phi (\textbf{x})$ is projected to the Poincaré ball via $\exp _{\textbf{0}}^c(\varvec{\mu }_\phi (\textbf{x}))$. The decoder’s first layer applies a gyroplane transformation^48,59^, which generalises affine maps to the Poincaré ball and allows hyperbolic coordinates to be mapped into Euclidean space. This is expressed in Eq. 21 where $\textbf{p}$ and $\textbf{a}$ are trainable parameters defining the orientation and offset of the gyroplane, $\textbf{z}$ is an input Poincaré ball vector to the layer, and $\langle \cdot ,\cdot \rangle$ denotes the Euclidean dot product.

21 $$\begin{aligned} d_p^c(\textbf{z}, H_{\textbf{a}, \textbf{p}}^c) = \frac{1}{\sqrt{c}} \sinh ^{-1} \left( \frac{2\sqrt{c}|\langle -\textbf{p} \oplus _c \textbf{z}, \textbf{a} \rangle |}{(1 - c\Vert -\textbf{p} \oplus _c \textbf{z}\Vert ^2) \Vert \textbf{a}\Vert } \right) \end{aligned}$$

For stability, group means are stored in Euclidean space and projected into $\mathbb {B}_c^d$ only when required, ensuring they remain inside the open ball and preventing geometric degeneracy. This design balances the representational advantages of hyperbolic geometry with the computational simplicity of Euclidean neural network layers, yielding a latent space that aligns naturally with the hierarchical structure of earthquake ground motion data.

In Poincaré ball geometry, distances grow exponentially away from the origin. If samples are embedded close to the edge of the ball, then this can lead to negative KL-divergence due to the approximation via probability densities. In such cases, it is highly plausible for the sample to be closer to the prior mean (density function $p_g(\cdot )$) than the posterior mean (density function $q_\phi (\cdot )$) (Eq. 18) such that $q_\phi (\textbf{z}\mid \textbf{x}) < p_g(\textbf{z})$, particularly true if the posterior variance is significantly small. With the KL-divergence approximated as the difference between the log-probability density functions, this leads to a negative term in the loss.

#### Group mean initialisation

In the hierarchical prior framework, each earthquake event is assigned a latent Gaussian distribution parameterised by its group mean $\varvec{\mu }_g$. During initial implementation, the hyper-level regularisation $L_{\textrm{reg,hyp}}$ (Eq. 14) included only the cohesion term, encouraging proximity between group means with similar event-level features. However, without an explicit dispersion component, optimisation exhibited degenerate behaviours that hindered latent space structuring.

Two intuitive initialisation strategies for $\varvec{\mu }_g$ were considered:

1. Random initialisation from the hyper-prior: Sampling from normal distribution $\mathcal {N}(\textbf{0}, \textbf{I})$ aligns with the target hyper-prior but results in an approximately uniform spread of feature-similar groups across the latent space. Cohesion forces then tend to pull these means toward the origin, while the hyper-prior KL term resists variance collapse. The outcome is minimal movement of group means and weak effective regularisation.
2. Zero initialisation: Setting all $\varvec{\mu }_g = \textbf{0}$ eliminates any initial separation, causing the cohesion term in $L_{\textrm{reg,hyp}}$ to vanish (all pairwise distances are zero) and trapping the system in a local optimum. In early epochs, the KL term can be trivially minimised by collapsing all sample embeddings near the origin, and variance inflation can occur artificially if only a few group means are pushed far away.

A practical compromise is to initialise group means close to the origin (variance $\sim 10^{-6}$), prompting movement while avoiding premature large-scale variance changes. However, this setup alone still leads to pathological clustering of group means without meaningful global structure. To address this, a dispersion term was introduced into $L_{\textrm{reg,hyp}}$, complementing cohesion with a repulsive force between dissimilar groups. This ensures that while similar groups remain close, unrelated groups are actively separated, producing a balanced, well-spread configuration.

**Fig. 12:**
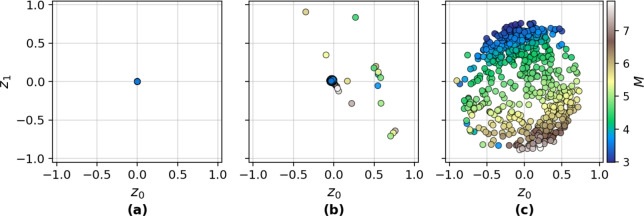
Effect of dispersion term on latent space. (**a**) The group means at epoch 0 (initialisation), they have been initialised using a normal distribution with variance $10^{-6}$. (**b**) The group means after 100 epochs without the dispersion term, there is a high concentration of group means at the origin. (**c**) The group means after 100 epochs with the dispersion term. Points have been colour coded based on magnitude *M*.

The effect of this dispersion term is illustrated in Fig. 12. Panel (a) shows the initialisation state at epoch 0, with all group means near the origin. Panel (b) depicts the configuration after 100 epochs without dispersion: group means remain tightly clustered, with minimal differentiation in the latent space. In contrast, panel (c) shows the configuration after 100 epochs with the dispersion term: group means are evenly distributed across the latent space, with a clear gradient in *M* (colour coded), indicating that event-level features are now meaningfully represented.

By enforcing both attraction among feature-similar groups and repulsion among dissimilar ones, this initialisation-plus-dispersion strategy yields a well-structured latent prior that supports the hierarchical embedding framework. Consequently, the zero-centred initialisation (approach 2) becomes viable when combined with dispersion regularisation.

#### HVAE neural network architecture

The encoder-decoder design in a VAE critically influences both latent space organisation and reconstruction fidelity^73^. In the proposed framework, the encoder must be sufficiently expressive to capture the multi-scale structure imposed by the hierarchical prior, dual-level regularisation, and hyperbolic geometry. Given this complexity, both encoder and decoder architectures are implemented as deep, high-capacity networks, with approximate mirroring of their layer configurations^73,74^ to promote symmetry in the encoding and decoding transformations.

Several architectural paradigms were evaluated prior to final selection. One-dimensional convolutional neural networks (1D-CNNs) were initially considered, motivated by prior success in modelling earthquake response spectra^40,75^ and by the analogy to image processing, where convolution preserves local structure. However, unlike images, where local smoothing typically retains semantic content, ground motion $S_a$ spectra exhibit fine-scale variations between adjacent *T* values that are seismologically significant, often linked to critical structural resonances. Convolutional downsampling can rapidly attenuate or distort these local features, risking physically unrealistic reconstructions. Consequently, convolutional layers were excluded. Long short-term memory (LSTM) layers were also explored, leveraging the analogy between period-indexed spectra and time-series sequences. In practice, however, LSTM-based encoders failed to converge to competitive reconstruction or latent regularisation performance.

The adopted architecture therefore comprises fully-connected (dense) layers organised into residual blocks^76^, enabling greater depth without degradation of gradient flow. This design supports both the high expressivity required by the encoder and the stability demanded by the decoder when mapping from the hyperbolic latent space. For the decoder, the first fully-connected layer is replaced with a Gyroplane layer to transform latent vectors from the Poincaré ball model into a Euclidean-compatible representation.

To parameterise network depth and width systematically, an architecture-generation function was implemented, taking as input the number of residual blocks *N* and a proportion vector $\textbf{P}$ controlling layer width relative to the input dimensionality. The first element of $\textbf{P}$ is fixed at 1.0 and subsequent values monotonically decrease to a minimum $>0.0$. This procedure produces *N* residual blocks, each containing two dense layers, followed by $|\textbf{P}|-N$ dense layers. The constraint $N < |\textbf{P}|$ ensures that block and post-block layers coexist. Algorithm 1 formalises this process.

Prior to any model training, the dataset underwent the filtering and KDE-based undersampling described in the Dataset sub-section. A stratified event-level split was then performed to prevent data leakage, allocating $72\%$ of events to training, $20\%$ to testing, and $8\%$ to validation. To support robustness assessment, the largest-*M* event was assigned to the test set, and the second-largest to the validation set.

The final encoder was generated with $N=1$ and $\textbf{P} = [1.0, 0.95, 0.9, 0.85]$, while the decoder used $N=2$ and $\textbf{P} = [1.0, 0.95, 0.9, 0.8, 0.7, 0.6, 0.5]$. Core hyperparameters were: latent dimensionality $d=2$, curvature $c=1.0$, group variance $\sigma _g^2=0.1$, dispersion base separation $s=2.0$, and loss weights of 30, 1, 50, 1000, and 10000.

Training proceeded for a minimum of 300 and a maximum of 500 epochs, with an early-stopping criterion applied independently to each loss term. Convergence was declared if, for either the training or validation set, the maximum relative loss reduction across all terms over the preceding 10 epochs fell below 0.001 of the loss at the eleventh previous epoch:

$$\max _{\ell \in \mathcal {L}} \frac{\textrm{mean}(\ell _{t-11:t-1} - \ell _{t-10:t})}{\ell _{t-11}} < 0.001.$$

This condition ensured that all loss components—including reconstruction, KL-divergences, and regularisation—were simultaneously stabilised before termination.

**Algorithm 1:**
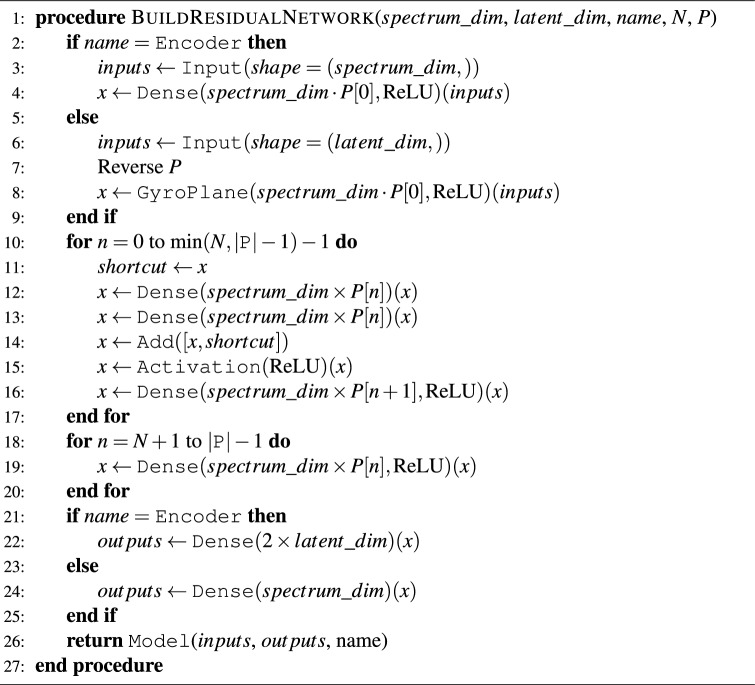
Build residual network

#### HVAE training configuration

The HVAE model was trained by simultaneously minimising five loss components: $L_{\text {recon}}$, $L_{\text {KL}}$, $L_{\text {reg}}$, $L_{\text {KL,hyp}}$, $L_{\text {reg,hyp}}$ using the parameter settings detailed in table 3.

**Table 3:** HVAE training configuration. All parameter settings that were used for training the HVAE.

Parameter	Value
Latent dimensions (*d*)	2
Negative curvature (*c*)	1.0
Reconstruction loss weight ($W_{\text {recon}}$)	30.0
KL-divergence loss weight ($W_{\text {KL}}$)	1.0
Regularisation loss weight ($W_{\text {reg}}$)	50.0
Hyper-KL-divergence loss weight ($W_{\text {KL,hyp}}$)	1000.0
Hyper-regularisation loss weight ($W_{\text {reg,hyp}}$)	10000.0
Group-level variance ($\varvec{\sigma }_g$)	0.1
Base dispersion (*s*)	2.0
Encoder residual blocks ($N_{\text {encoder}}$)	1
Encoder weight proportion vector ($\textbf{P}_{\text {encoder}}$)	[1, 0.95, 0.9, 0.85]
Decoder residual blocks ($N_{\text {decoder}}$)	2
Decoder weight proportion vector ($\textbf{P}_{\text {decoder}}$)	[1.0, 0.95, 0.9, 0.8, 0.7, 0.6, 0.5]
Minimum epochs	300
Maximum epochs	500

#### Downstream application model designs

The application of the HVAE within and early warning system and for ground motion time history simulation required the design and implementation of an FFNN and CVAE respectively.

The FFNN architecture utilised ten fully-connected layers with the number of neurons per layer monotonically decreasing from 60 to 30, followed by the two-neuron full-connected output layer. Input features were log-transformed prior to training, the loss function was simply the average Poincaré ball distance (Eq. 18) between the known $\textbf{z}$ for and the predicted $\mathbf {{\hat{z}}}$, and the model was trained for 500 epochs.

The CVAE architecture similarly utilised seven full-connected layers for the encoder with the number of neurons per layer monotonically decreasing from 50 to 20, followed by a ten-dimensional latent space. The decoder network had a similar architecture with seven full-connected layers and number of neurons per layer monotonically decreasing from 50 to 10, followed by the two-neuron fully-connected output layer. The model was trained by minimising the average Poincaré ball distance (Eq. 18) between the known $\varvec{\mu }_\phi (\textbf{x})$ and a predicted $\varvec{{\hat{\mu }}}_\psi (\textbf{x})$ for each sample $\textbf{x}$, where $\psi$ are the parameters of the CVAE. All three models (HVAE, FFNN, and CVAE) can be viewed and assessed through the supporting code provided in the Code Availability sub-section.

## Data Availability

The ground motion records used in this study are available from the PEER NGA-West2 Ground Motion Database72, accessible at https://ngawest2.berkeley.edu

## Code availability

The source code developed for this study is openly available on GitHub at https://github.com/jfayaz/HVAE. The provided implementation was developed using Python version 3.10.6 and TensorFlow version 2.16.1.
